# qcCHIP: an R package to identify clonal hematopoiesis variants using cohort-specific data characteristics

**DOI:** 10.1093/bioinformatics/btaf522

**Published:** 2025-09-17

**Authors:** Xiang Liu, Yi-Han Tang, James Blachly, Stephen Edge, Yasminka A Jakubek, Martin McCarter, Abdul Rafeh Naqash, Kenneth G Nepple, Afaf Osman, Matthew J Reilley, Gregory Riedlinger, Bodour Salhia, Bryan P Schneider, Craig Shriver, Michelle L Churchman, Robert J Rounbehler, Jamie K Teer, Nancy Gillis, Mingxiang Teng

**Affiliations:** Department of Biostatistics and Bioinformatics, H. Lee Moffitt Cancer Center and Research Institute, Tampa, FL 33612, United States; Department of Biostatistics and Bioinformatics, H. Lee Moffitt Cancer Center and Research Institute, Tampa, FL 33612, United States; Department of Cancer Epidemiology, H. Lee Moffitt Cancer Center and Research Institute, Tampa, FL 33612, United States; The Ohio State University Comprehensive Cancer Center, Columbus, OH 43210, United States; Roswell Park Comprehensive Cancer Center, Buffalo, NY 14203, United States; University of Kentucky Markey Cancer Center, Lexington, KY 40536, United States; University of Colorado Cancer Center, Aurora, CO 80045, United States; University of Oklahoma Stephenson Cancer Center, Oklahoma City, OK 73104, United States; University of Iowa Holden Comprehensive Cancer Center, Iowa City, IA 52242, United States; University of Utah Huntsman Cancer Institute, Salt Lake City, UT 84112, United States; University of Virginia Comprehensive Cancer Center, Charlottesville, VA 22903, United States; Rutgers Cancer Institute, New Brunswick, NJ 08901, United States; Norris Comprehensive Cancer Center, University of Southern California, Los Angeles, CA 90089, United States; Indiana University Simon Comprehensive Cancer Center, Indianapolis, IN 46202, United States; Murtha Cancer Center, Bethesda, MD 20814, United States; Aster Insights, Hudson, FL 34667, United States; Aster Insights, Hudson, FL 34667, United States; Department of Biostatistics and Bioinformatics, H. Lee Moffitt Cancer Center and Research Institute, Tampa, FL 33612, United States; Department of Cancer Epidemiology, H. Lee Moffitt Cancer Center and Research Institute, Tampa, FL 33612, United States; Department of Biostatistics and Bioinformatics, H. Lee Moffitt Cancer Center and Research Institute, Tampa, FL 33612, United States

## Abstract

**Summary:**

Clonal hematopoiesis (CH) is a molecular biomarker associated with various adverse outcomes in both healthy individuals and those with underlying conditions, including cancer. Detecting CH usually involves genomic sequencing of individual blood samples followed by robust bioinformatics data filtering. We report an R package, qcCHIP, a bioinformatics pipeline that implements permutation-based parameter optimization to guide quality control filtering and cohort-specific CH identification. We benchmark qcCHIP under various data settings, including different sequencing depths, ranges of cohort sizes, with and without normal-tumor paired samples, and across different cancer types. We show that qcCHIP allows users to customize analysis needs to generate CH calls based on cohort-specific data characteristics.

**Availability and implementation:**

qcCHIP R package is freely accessible at GitHub https://github.com/tenglab/qcCHIP and DOI: 10.5281/zenodo.16421861.

## 1 Introduction

Clonal hematopoiesis (CH) is a molecular biomarker associated with various adverse outcomes, including increased risks for hematologic malignancies and reduced overall survival in both healthy individuals and patients with cancer (Genovese *et al.* 2014, Jaiswal *et al.* 2014, Coombs *et al.* 2017, Walsh *et al.* 2022). CH is characterized by recurrent somatic mutations in the blood or bone marrow of individuals without overt hematologic abnormalities. These mutations typically occur at low variant allele frequencies (VAFs, <20%) within a heterogeneous population of hematopoietic cells, making their detection challenging. Unlike germline or tumor-derived somatic mutations, CH mutations lack clonal dominance and may resemble sequencing artifacts. Detection of CH is further complicated by the absence of matched normal tissue and the need to distinguish CH from both inherited and tumor-derived somatic variants.

**Figure 1.**
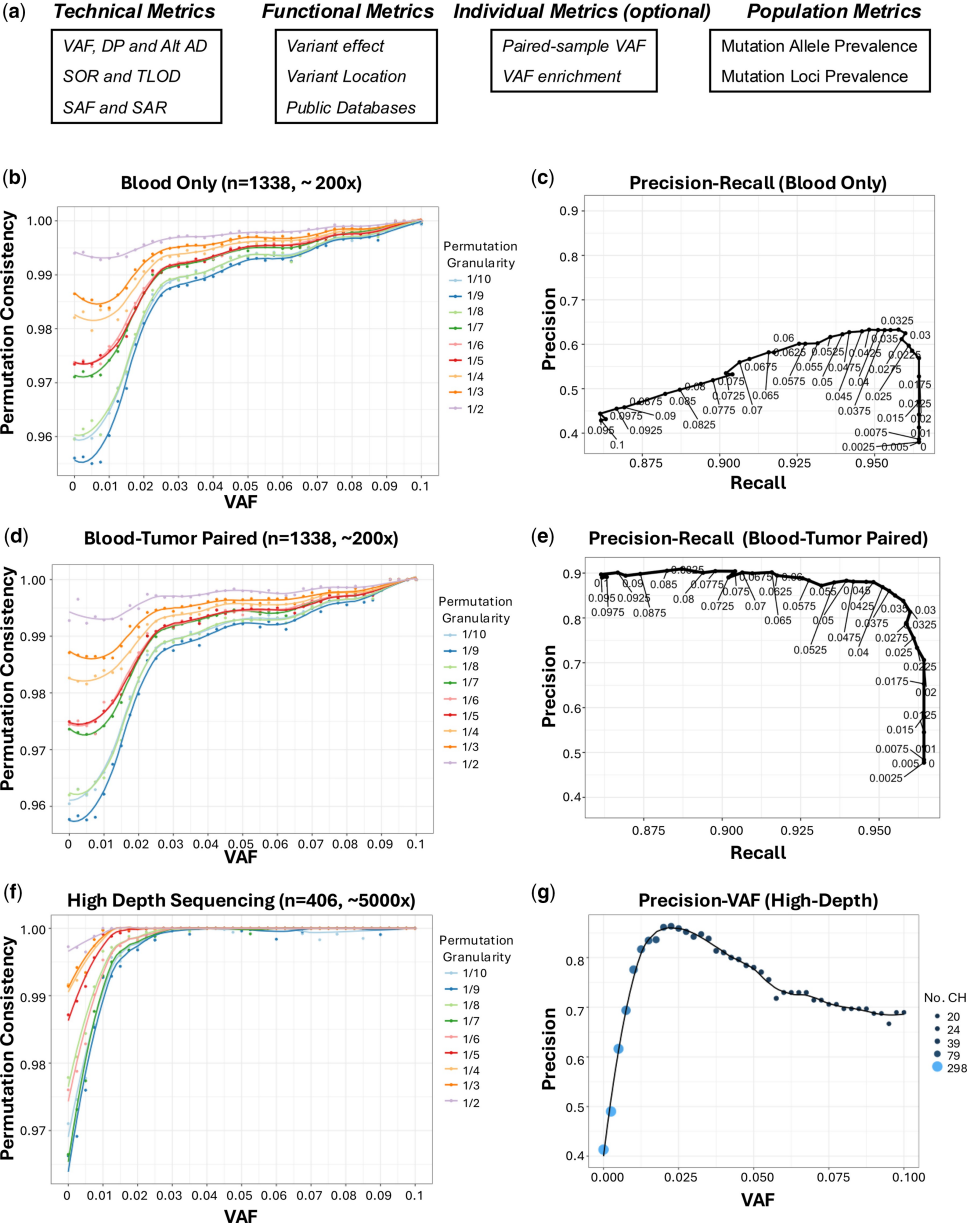
Permutation-based approach to optimize clonal hematopoiesis (CH) quality control metric cutoffs. (a) Quality metrics to filter CH mutations by qcCHIP. (b) Permutation analysis and (c) precision and recall at different variant allele frequency (VAF) cutoffs using blood-only whole exome sequencing data from the ORIEN breast cancer cohort. Permutation consistency is the proportion of CH calls from the full cohort that are also called in the permuted subsets. Each point represents average consistency over 100 permutations. Colors indicate different permuted sample sizes. (d) Permutation analysis and (e) precision and recall at different VAF cutoffs using blood-tumor paired samples for the same breast cancer cohort. (f) Permutation analysis and (g) precision-VAF curves using blood-only ultra-high-depth targeted exon sequencing from an independent cohort of patients with breast cancer. Minor effects on recall are showed in Fig. 2, available as supplementary data at *Bioinformatics* online. The point sizes represent the number of common CH mutations between qcCHIP identification and manually curated CH mutations.

Identifying CH usually involves targeted or whole-exome DNA sequencing of peripheral blood samples followed by bioinformatics analysis involving mutation calling and rigorous quality filtering (Coombs *et al.* 2018, Chan *et al.* 2024). For quality filtering, the state-of-art bioinformatics pipelines, including those applied in our previous studies (Gillis *et al.* 2017, Bolton *et al.* 2019, Peres *et al.* 2022, Gillis *et al.* 2024), focus on reducing the effects of technical artifacts (e.g. based on VAF, sequencing coverage, etc.) and functional uncertainties (e.g. synonymity, public knowledge, etc.); however, this ignores potential artifacts caused by cohort-specific characteristics. For instance, blood samples from patients with cancer can be confounded by tumor cell-free DNA, or patients with lung cancer may have a distinct pool of CH alterations compared to healthy individuals or patients with breast cancer (Coombs *et al.* 2017, Coombs *et al.* 2018). Ignoring these cohort-specific artifactual features could lead to false CH calls. Moreover, existing quality filtering approaches often rely on arbitrary parameter thresholds, such as VAF cutoff ≥ 0.02 (Jaiswal *et al.* 2014, Xie *et al.* 2014, Steensma *et al.* 2015), with limited evidence to support their applicability across diverse study cohorts with variable sequencing data. The ArCH tool provides sensitivity to detect lower VAF (<0.02) and enables a full spectrum of tasks including mutation calling, annotation, and quality filtering (Chan *et al.* 2024), yet it does not allow users to optimize parameters based on cohort-specific characteristics (Fig. 1, available as supplementary data at *Bioinformatics* online). Here, we introduce an easy-to-use R package, qcCHIP, that implements cohort-specific quality control and permutation-based optimization for parameter selection in CH identification. We benchmark the performance of CH calling based on various sequencing protocols and sample cohorts.

## 2 Methods

### 2.1 Mutation calls as the package input

qcCHIP focuses on quality control of CH candidate mutations (i.e. single-nucleotide variants and small insertions and deletions). It adopts single-sample mutation calls that are stored in the standard VCF format. We chose VCF format as input because it is widely output by common mutation callers, e.g. Mutect2 (McKenna *et al.* 2010), regardless of sequencing protocols and platforms. By default, mutation calls from blood samples are required while mutations from other confounding samples (e.g. paired tumor samples) are optional. As qcCHIP allows filtering using variant functional effects, the input VCF files are recommended to be annotated by software such as ANNOVAR (Wang *et al.* 2010).

### 2.2 Cohort-specific metrics to control CH filtering

Four groups of cohort-specific quality metrics are used to filter CH candidates: technical-, functional-, individual- and populational-related metrics (Fig. 1a). For technical metrics, we set cutoffs on technical parameters to optimize reliable mutation calling and to remove germline mutations. These parameters include VAF, read depth (DP), strand bias based on strand odds ratio (SOR), alternative allele depth (Alt_AD), forward/reverse strand reads (SAF/SAR), and likelihood of variant existing using the tumor log odds (TLOD). In brief, a reliable somatic mutation should hold confident VAF and read coverage but minimized strand bias. For functional metrics, we evaluate existing evidence to remove likely non-functional mutations. We collected evidence including variant functional effect (e.g. annotated by ANNOVAR), variant location (e.g. repetitive regions), and prevalence in gnomAD (Karczewski *et al.* 2020), dbGAP (Tryka *et al.* 2014), and COSMIC (Tate *et al.* 2019) databases. In brief, a functional CH mutation should not be synonymous and not frequently reported in population-level databases of germline variants but may be observed in cancer-specific databases. The technical and functional metrics were selected based on our established experience and others’ (Gillis *et al.* 2017, Coombs *et al.* 2018, Vlasschaert *et al.* 2023). For individual metrics, we recruit non-blood samples from the same individuals to eliminate individual mutation biases. For instance, in typical whole exome sequencing (WES) studies, mutations appearing in both blood and paired-tumor samples at a much lower VAF in blood suggests that blood mutation calls are confounded by tumor cell-free DNA. Mutations at similar VAF in blood and tumor may be germline and not relevant to CH calling. Conversely, mutations with significant enrichment of VAF in blood compared to paired samples are considered as potentially true CH. Individual metrics are optional by qcCHIP given that not all CH studies perform sequencing on more than blood samples. For population metrics, since individual CH alterations are rare, we introduce prevalence-based metrics to control CH occurrences in a given cohort under the rationale of that mutations holding higher frequency than expected in a cohort are unlikely true CH mutation events. For a given mutation, its prevalence is examined at both allele and locus levels. To avoid potential confounding with other metrics, population metrics are implemented at the beginning of the pipeline. The default values of individual metrics are explained in the R package.

### 2.3 Permutation-based guidance to select cohort-specific parameter values

By default, parameters of the above metrics are set based on published evidence. For example, the default VAF is ≥0.02 since WES studies found cutoff at 2% generates good balance between the reliability and sensitivity of CH detection. However, ultra-high-depth error-corrected sequencing (e.g. depth ≥ 5000×), can confidently detect mutations with VAF < 0.02 (Young *et al.* 2016). Similarly, the commonly used DP >20 cutoff has different implications for WES (typically 100–200×) versus high-depth sequencing (1000–5000×). Also, filtering based on mutation prevalence (e.g. 10%) to remove likely artifactual variants provides varied reliability between small (e.g. *n* < 50) and larger cohorts (*n* > 1000). To address these challenges, we developed a permutation-based strategy to inform parameter selection, tailored to cohort-specific characteristics. We hypothesized that optimal parameters generate less varied CH calls between the whole cohort and permuted subsets of the same cohort. For a given cohort, we permuted its subsets at different proportions (i.e. 1/2, 1/3, …0.1/10) of the full cohort. We called CH based on the full cohort and the subsets of samples. We then pooled the calls from subset samples in each permutation. Consistency was measured by the percentages of the common and unique CH calls between the full cohort and permuted subsets. In Fig. 1b, d, and f, we demonstrate the permutation analysis on VAF selection using different cohorts of samples from patients with breast cancer. Consistency was compared at different VAF values ranging from 0 to 0.1. The consistency-VAF curves at different permutation sizes all show inflection points at which the VAF cutoffs give relatively saturated consistency. In addition, consistency typically declined as the size of the permuted subset decreased at fixed VAF cutoffs, showing that smaller sample sizes require higher VAF thresholds to maintain consistency. For example, in the 200× blood-only cohort (*n* = 1338) (Fig. 1b), the 1/2 subset achieved high consistency at a VAF of 0.02, whereas the 1/10 subset reached its optimal consistency at a higher VAF of 0.025. In contrast, using data with a sequencing depth of 5000× (*n* = 406), a low VAF at 0.01 yielded saturated consistency across the 1/2 to 1/5 subsets. Using manually curated CH data from the same cohorts, we further demonstrated that the permutation-based VAF selection achieved optimal precision and/or recall, with comparable inflection points on the precision and recall curves (Fig. 1c, e, g and Fig. 2, available as supplementary data at *Bioinformatics* online). Manually curated CH went through technical examination in the IGV browser and functional evaluation with public knowledge and databases. CH comparisons between qcCHIP (under default parameter settings) and the manual references are summarized in Table 1, available as supplementary data at *Bioinformatics* online. Results were consistent with existing studies, in that hematologic malignancy-related genes such as *DNMT3A* and *TET2* were the most enriched with CH mutations (Genovese *et al.* 2014, Jaiswal *et al.* 2014, Coombs *et al.* 2018, Young *et al.* 2016) (Figs 3–5, available as supplementary data at *Bioinformatics* online). These findings highlight the efficacy of the permutation-based approach. We observed similar patterns of inflection points and permutation consistency in other cohorts (lung and colorectal cancers, Figs 6 and 7, available as supplementary data at *Bioinformatics* online) and stratified by other parameters (i.e. cohort mutation prevalence, DP, SOR, and SAF/SAR) (see Figs 8–24, available as supplementary data at *Bioinformatics* online). In summary, permutation-based analysis facilitates the tailored determination of parameters based on the unique sequencing, sample size, and sample type characteristics of each cohort.

### 2.4 R Package implementation

The R package is implemented with three functions: *vcf2input*, *CHIPfilter* and *qcCHIP* (Fig. 25, available as supplementary data at *Bioinformatics* online). The *vcf2input* function merges and simplifies mutation VCF files to generate input for *CHIPfilter* and *qcCHIP*. This saves computing resources and allows the package to fit large cohort studies on personal computers. VCF files can be either raw generated by mutation callers or annotated by tools such as ANNOVAR. The *CHIPfilter* function filters mutations and identifies CHIP candidates using the four metric types discussed above (technical, functional, individual, and population-based). *CHIPfilter* can be run under two modes: single-sample (i.e. blood or bone marrow sample) and paired-sample (e.g. paired blood/bone marrow and non-blood control samples). Paired-sample mode is recommended if paired samples are available such as those from cancer studies. Metric parameters are tunable with default values set to fit typical WES datasets. For a given cohort, it is recommended to first apply the *qcCHIP* function to perform permutation analysis and guide the determination of optimal parameters. Currently, the *qcCHIP* function provides evaluation for five metrics: VAF, cohort mutation prevalence, DP, SOR, and SAF/SAR. It generates plots to visualize the performance of CH identification stratified by the five metrics. The permutation analysis can be set with customized metric ranges and steps. The runtime and memory cost of permutation steps at varied sample sizes and subset groups are listed in Fig. 26, available as supplementary data at *Bioinformatics* online.

## 3 Benchmark and use cases

We applied qcCHIP to four independent datasets across cancer cohorts and sequencing depths. These cohorts reflect varied cohort sizes, cancer groups, sequencing depths, and paired/non-paired studies. Three cohorts are based on WES sequencing from the ORIEN network (Wang *et al.* 2024), covering blood and paired tumor samples from breast (*n* = 1338), lung (*n* = 567), and colorectal (*n* = 1132) cancers. Breast, lung, and colorectal cancers are associated with adverse risks from CH (Comen *et al.* 2020, Nguyen *et al.* 2021, Liu *et al.* 2025). The fourth cohort is targeted ultra-high-depth (∼5000X) error-corrected sequencing of blood from 406 samples from patients with breast cancer. For the ORIEN cohorts both blood-only (Fig. 1b) and blood-tumor paired analysis were performed (Fig. 1d). For ultra-high-depth sequencing cohort, blood-only analysis was performed (Fig. 1f). Five metrics were demonstrated including VAF, cohort mutation prevalence, DP, SOR and SAF/SAR.

For VAF selection, inflection points were clearly observed on curves involving permutation consistency, precision, and recall, regardless of whether there were paired tumor samples (Fig. 1 and Figs 2, 6, and 7, available as supplementary data at *Bioinformatics* online). For ORIEN cohorts, the optimal VAF by permutation analysis is around 0.02 to 0.025, consistent with existing knowledge on CH calling with WES data (Jaiswal *et al.* 2014, Xie *et al.* 2014, Steensma *et al.* 2015). The VAF inflection value is consistent between blood-only and blood-tumor paired permutation analysis. In contrast, the inflection value of VAF is lower (approximately 0.01) for the ultra-high-depth sequencing, indicating that deeper sequencing allows a more relaxed VAF cutoff to maintain similar consistency. In a given cohort, smaller subsets require higher VAF cutoffs to achieve the same consistency, highlighting the impact of cohort size on VAF determination. Overall, the precision and recall curves show similar optimal VAFs across cohorts, with the exception of the ultra-high-depth sequencing cohort, where recall is high regardless of cutoff.

For cohort mutation prevalence selection, we observed a fluctuated but overall increased consistency across prevalence values ranging from 0 and 0.15 (Figs 8–12, available as supplementary data at *Bioinformatics* online). The consistency decreases when permutation sample size gets small, similar to what was observed in the analysis of VAF. We recommend 0.1 as the default prevalence cutoff, although a cutoff between 0.05 and 0.1 yields similar performance, as indicated by the precision and recall. The observed fluctuated consistency was caused by a few mutations with high prevalence but low VAF (<0.05) in the studied cohorts. If permutation resulted in imbalance of these mutations across subsets, discrepancies between results of the whole cohort and the subsets could be observed. Since these mutations usually hold low VAFs, we suggest a higher VAF cutoff (e.g. 0.05 for WES) to reduce fluctuations when permuting mutation prevalences. We show that the patterns of permutation curves stay consistent between VAF = 0.02 and VAF = 0.05 (panels a-d in Figs 8–11, available as supplementary data at *Bioinformatics* online). To test the robustness of permutation with larger sample sizes, we combined the three cancer cohorts and performed permutation analysis on the combined cohort. The results indicate that the combined cohort results in a similar mutation prevalence cutoff as the individual cancer cohorts (Fig. 11, available as supplementary data at *Bioinformatics* online).

For DP selection, we observed an inflection value around 20 (Figs 13–16, available as supplementary data at *Bioinformatics* online). Higher cutoffs give decreased consistency but increased precision. These patterns are consistent between blood-only and blood-tumor paired analysis across the ORIEN cohorts. This suggests that high DP is more sensitive to permutation, likely due to inconsistent DPs at the same loci across samples. DP selection had minimal effect on recall of the ORIEN cohorts and precision/recall of the ultra-high-depth cohort where most CH candidates were highly covered. For SOR selection, we aimed to identify an upper bound value to minimize strand bias for CH filtering (Figs 17–20, available as supplementary data at *Bioinformatics* online). We found its inflection value at around 2 to 3 across the studied cohorts. Higher SOR cutoffs significantly decreased permutation consistency and precision with limited effect on recall. For SAF/SAR, we aimed to determine a lower bound value to inform the minimal number of reads to maximize CH confidence (Figs 21–24, available as supplementary data at *Bioinformatics* online). We found the inflection value around 3 to 5 in the three ORIEN cohorts. Lower SAF/SAR cutoffs significantly decreased permutation consistency, precision, and recall. The ultra-high-depth cohort did not show a converging consistency with SAF/SAR, likely due to the high coverage for most mutations.

## 4 Conclusion

We presented a permutation-based approach to guide parameter optimization in CH identification from blood or bone marrow sequencing datasets. We demonstrated the effects of parameter selection (i.e. VAF, cohort mutation prevalence, DP, SOR and SAF/SAR) using permutation consistency, precision, and recall. We show that optimal parameters vary across datasets and cohort sizes. This is the first tool for evidence-based, cohort-specific quality control in CH mutation calling, implemented as a user-friendly R package for parameter optimization and visualization.

## Supplementary Material

btaf522_Supplementary_Data

## Data Availability

The data underlying this article were provided by Aster Insights in collaboration with the Oncology Research Information Exchange Network. Data will be shared on request to the corresponding author with permission of Aster Insights.
