# Supplementary material for: qcCHIP: an R package to identify clonal hematopoiesis variants using cohort-specific data characteristics
Source: Bioinformatics. 2025 Sep 17;41(9):btaf522. doi: 10.1093/bioinformatics/btaf522 (PMC12466928; doi:10.1093/bioinformatics/btaf522)
Supplement: btaf522_Supplementary_Data [file btaf522_supplementary_data.zip › Supplementary_Figs_revise.pdf]

Tool	Variant calling	Variant annotation	Variant filtering	Filtering parameter optimization
ArCH	✓	✓	✓	X
qcCHIP	X	X	✓	✓

Supplementary Figure 1. Main functional modules of ArCH and qcCHIP tools.

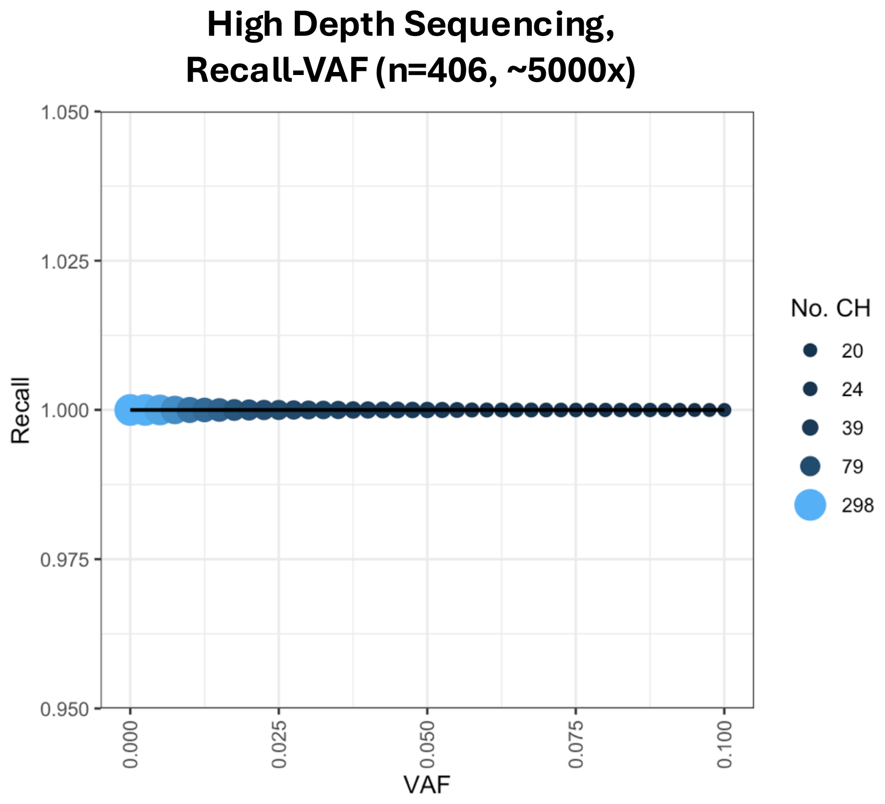

Supplementary Figure 2. Recall-VAF curve based on blood samples of the ultra-high-depth sequencing breast cancer cohort.

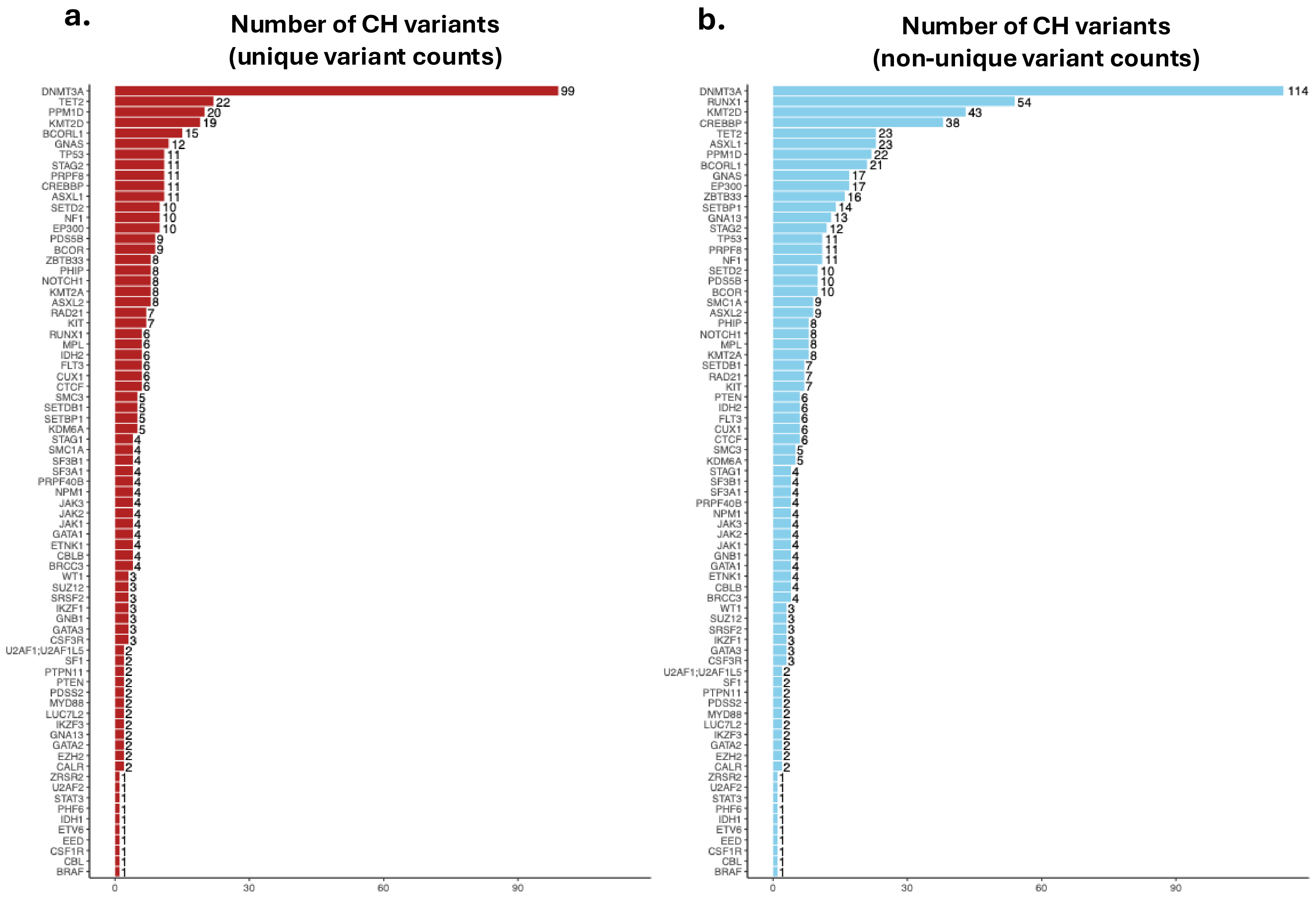

Supplementary Figure 3. CH occurrences in hematologic malignancy-related genes identified in the ORIEN breast cancer cohort using blood-tumor samples. **a).** The same variants observed in multiple samples were counted once. **b).** The same variants observed in multiple samples were counted each time they appeared.

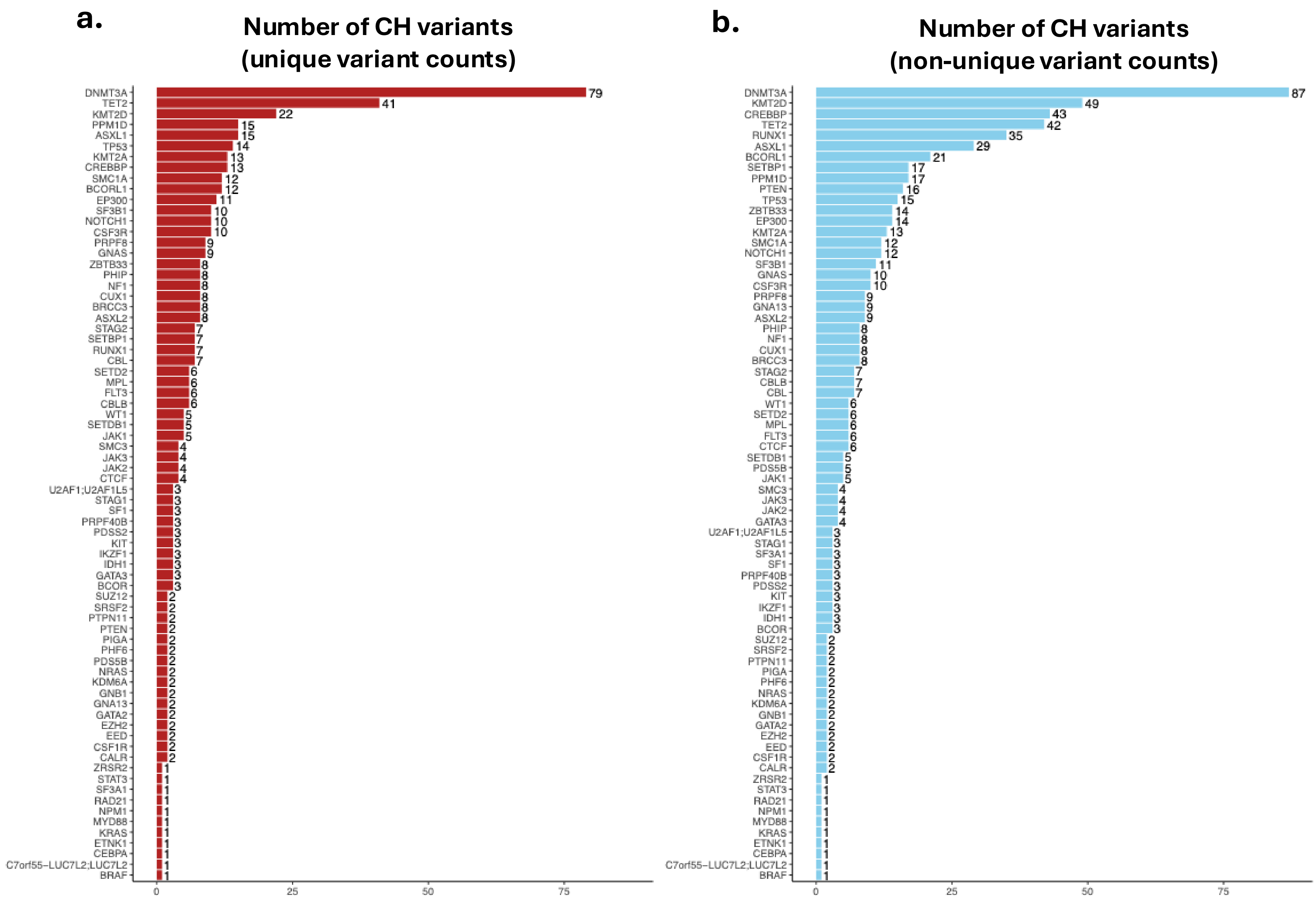

Supplementary Figure 4. CH occurrences in hematologic malignancy-related genes identified from ORIEN colorectal cancer cohort using blood-tumor samples. **a).** The same variants observed in multiple samples were counted once. **b).** The same variants observed in multiple samples were counted each time they appeared.

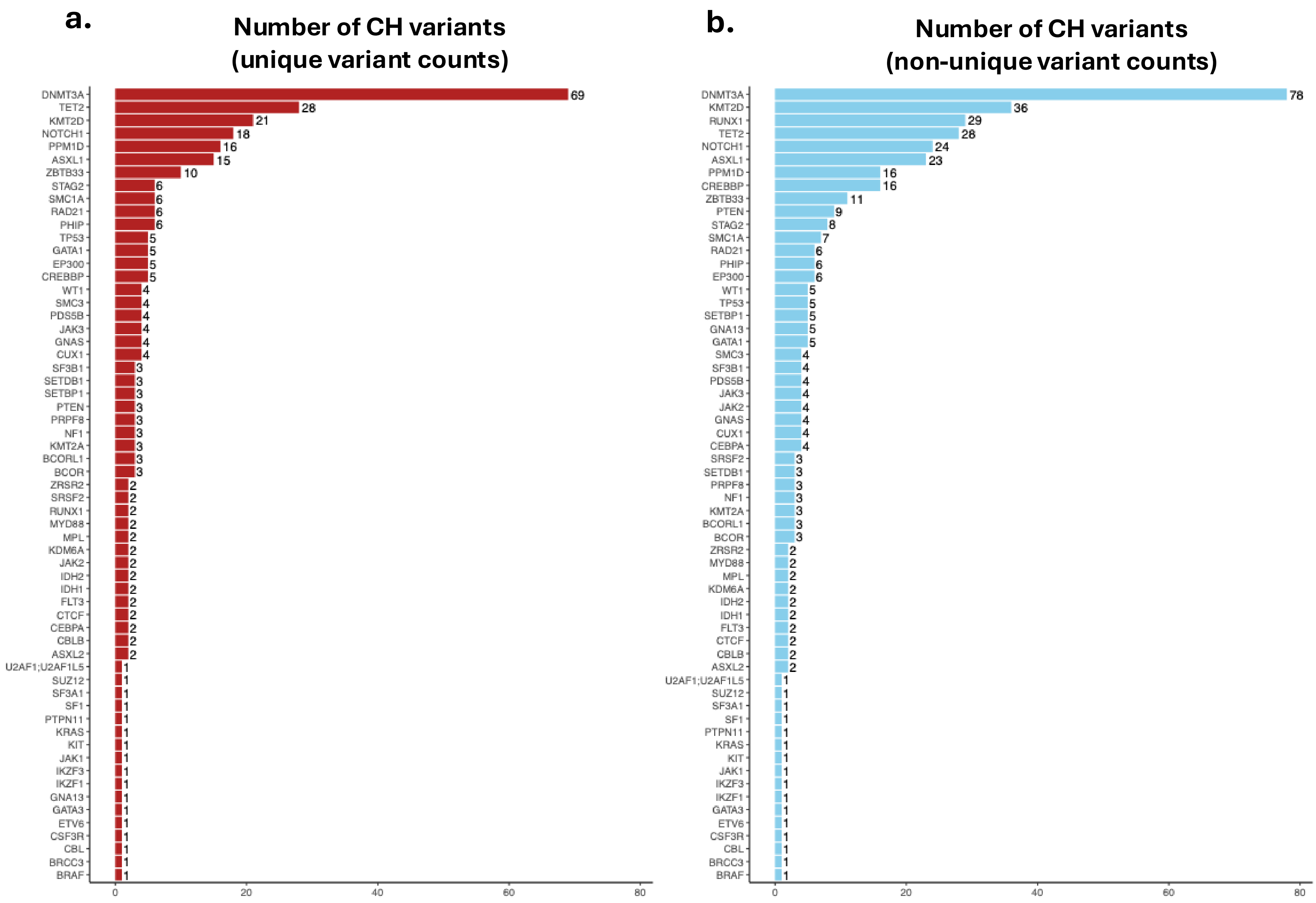

Supplementary Figure 5. CH occurrences in hematologic malignancy-related genes identified from ORIEN lung cancer cohort using blood-tumor samples. **a).** The same variants observed in multiple samples were counted once. **b).** The same variants observed in multiple samples were counted each time they appeared.

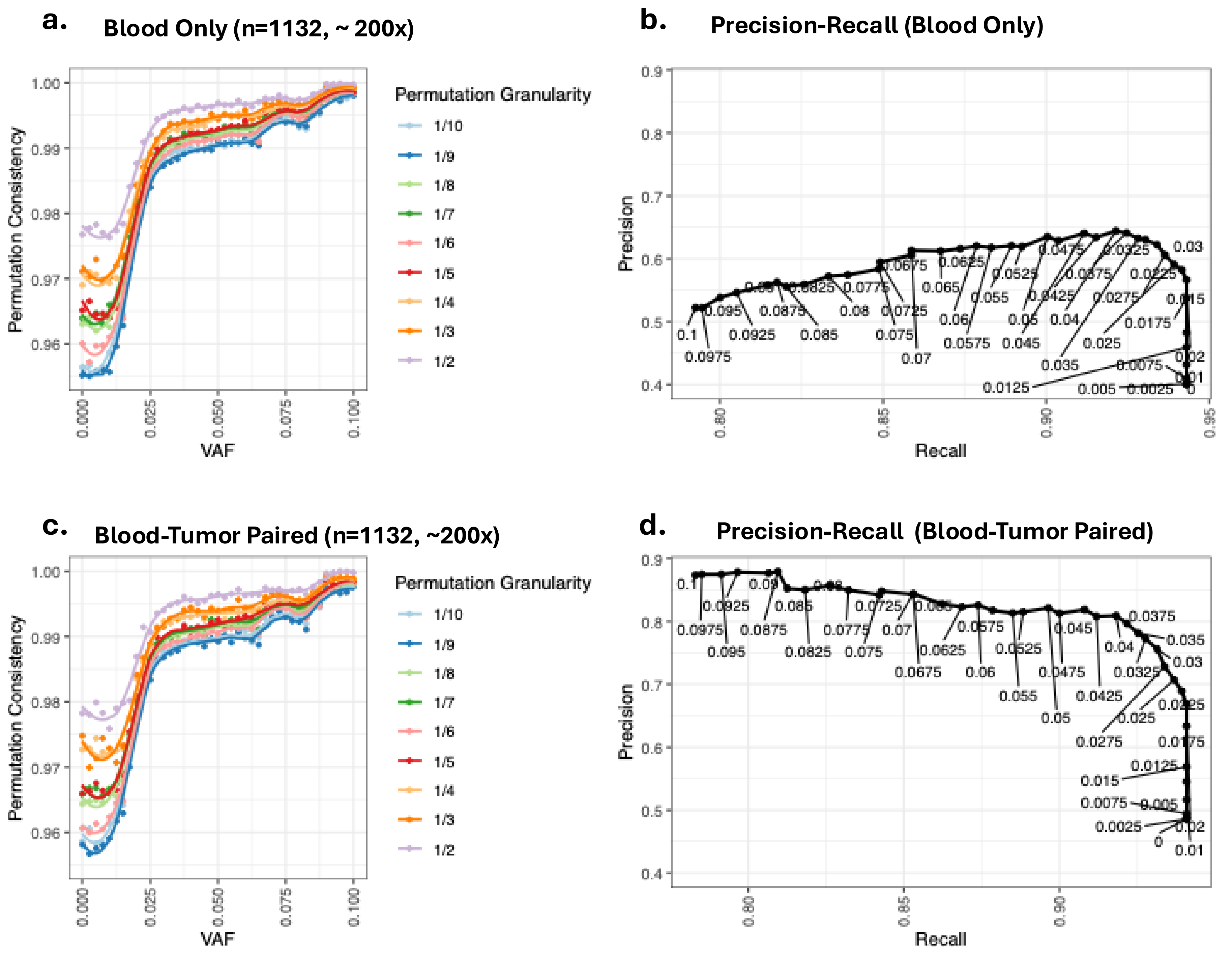

Supplementary Figure 6. VAF permutation analysis of the ORIEN colorectal cancer cohort. **a).** Permutation consistency using blood-only samples. **b).** Precision and recall at different VAF cutoffs using blood-only samples. **c).** Permutation consistency using blood-tumor paired samples. **d).** Precision and recall at different VAF cutoffs using blood-tumor paired samples

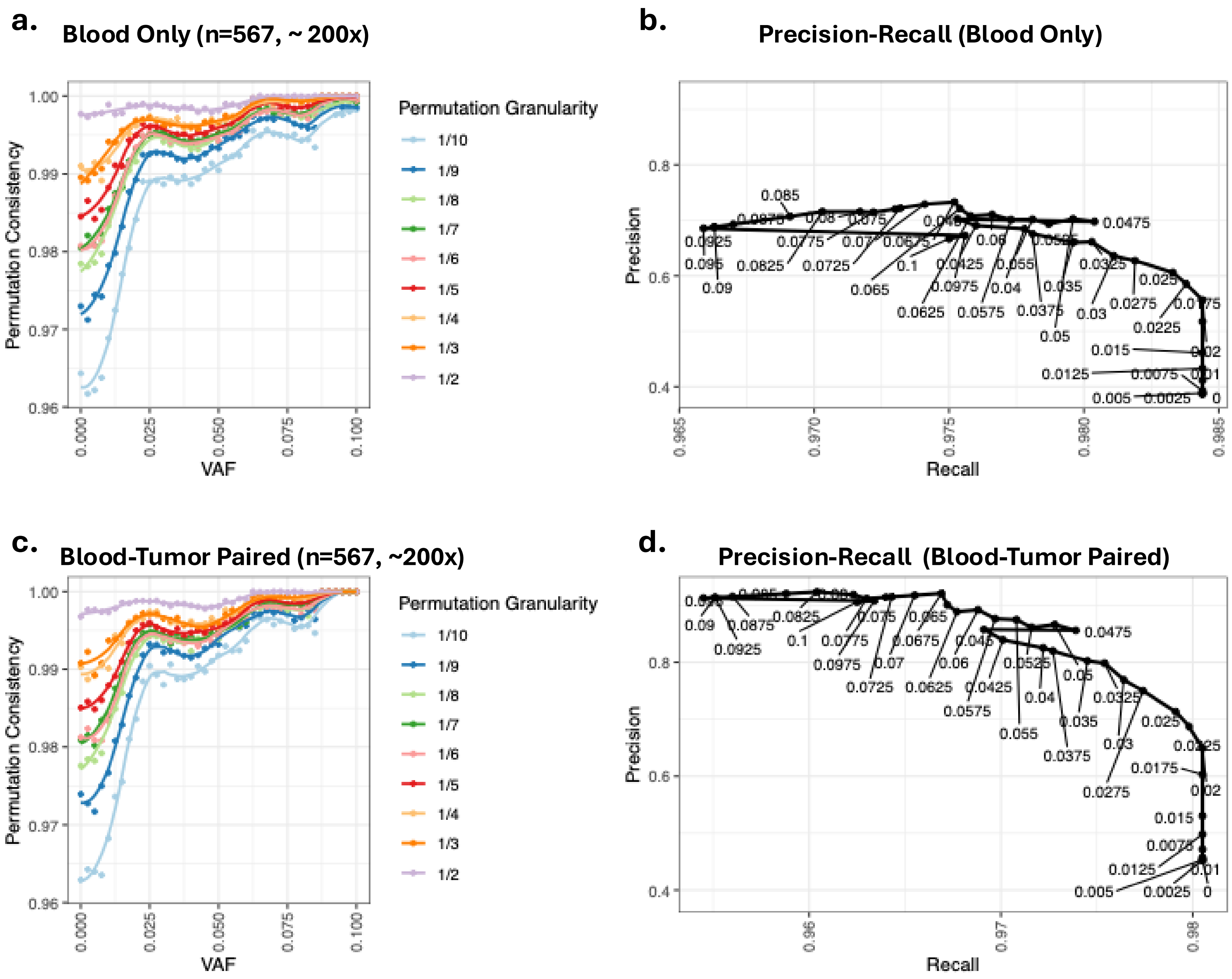

Supplementary Figure 7. VAF permutation analysis of the ORIEN lung cancer cohort. **a).** Permutation consistency using blood-only samples. **b).** Precision and recall at different VAF cutoffs using blood-only samples. **c).** Permutation consistency using blood-tumor paired samples. **d).** Precision and recall at different VAF cutoffs using blood-tumor paired samples

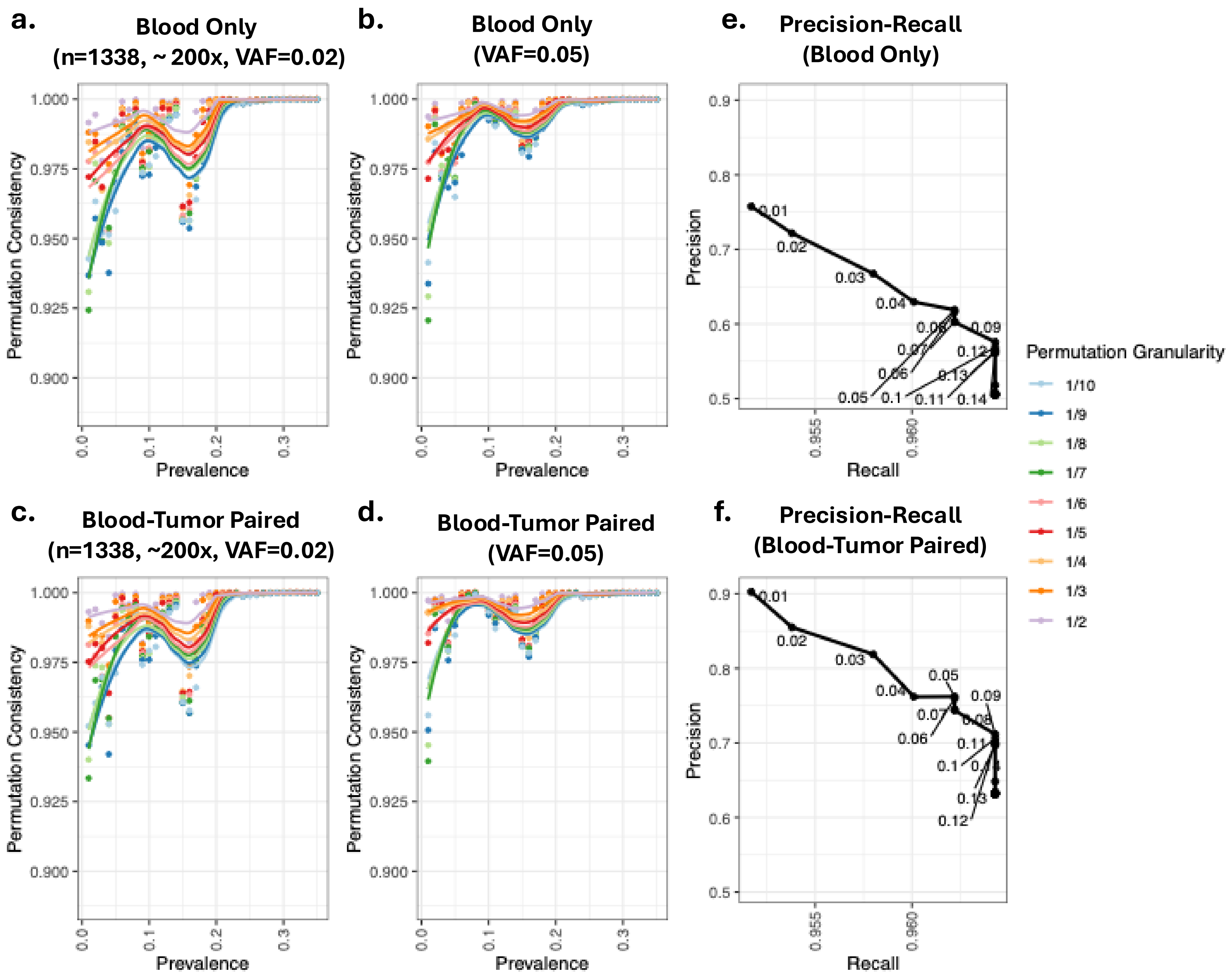

Supplementary Figure 8. Prevalence permutation analysis in the ORIEN breast cancer cohort. **a)**. Permutation consistency using blood-only samples at VAF=0.02 and **b)**. VAF=0.05. **c)**. Permutation consistency using blood-tumor paired samples at VAF=0.02 and **d)**. VAF=0.05. **e)**. Precision and recall at different prevalence cutoffs using blood-only samples and **f)**. blood-tumor paired samples.

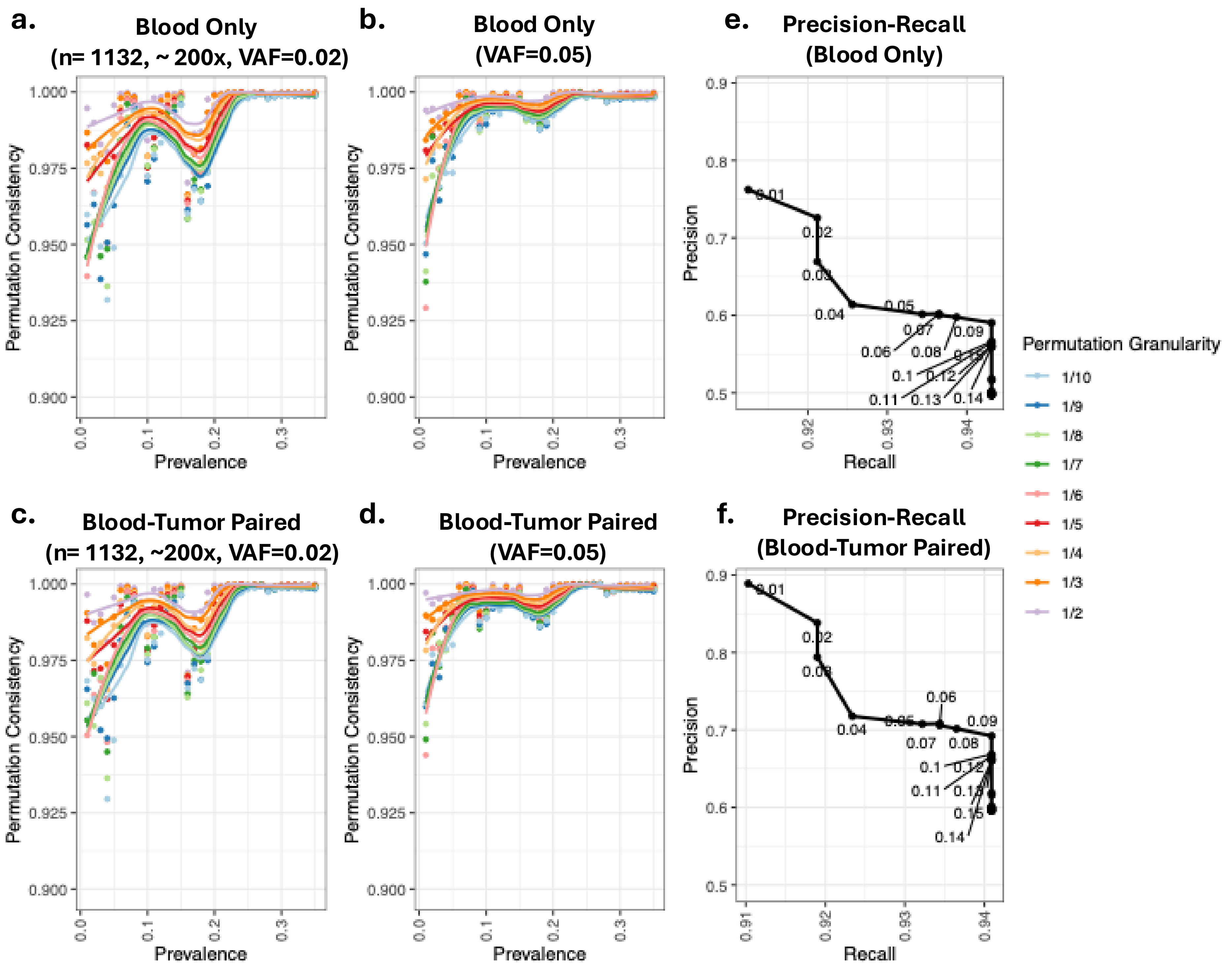

Supplementary Figure 9. Prevalence permutation analysis in the ORIEN colorectal cancer cohort. **a)**. Permutation consistency using blood-only samples at VAF=0.02 and **b)**. VAF=0.05. **c)**. Permutation consistency using blood-tumor paired samples at VAF=0.02 and **d)**. VAF=0.05. **e)**. Precision and recall at different prevalence cutoffs using blood-only samples and **f)**. blood-tumor paired samples.

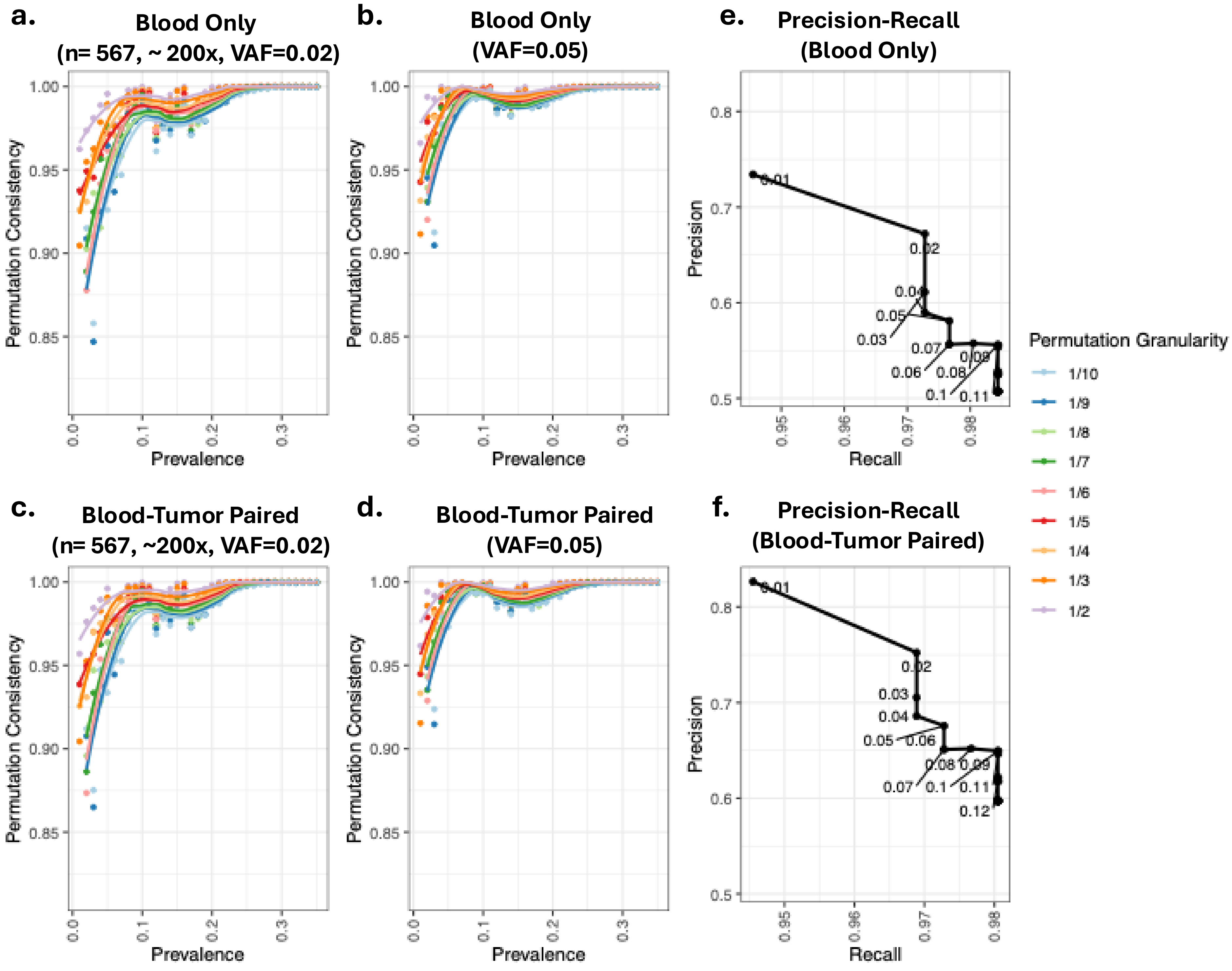

Supplementary Figure 10. Prevalence permutation analysis in the ORIEN lung cancer cohort. **a).** Permutation consistency using blood-only samples at VAF=0.02 and **b).** VAF=0.05. **c).** Permutation consistency using blood-tumor paired samples at VAF=0.02 and **d).** VAF=0.05. **e).** Precision and recall at different prevalence cutoffs using blood-only samples and **f).** blood-tumor paired samples.

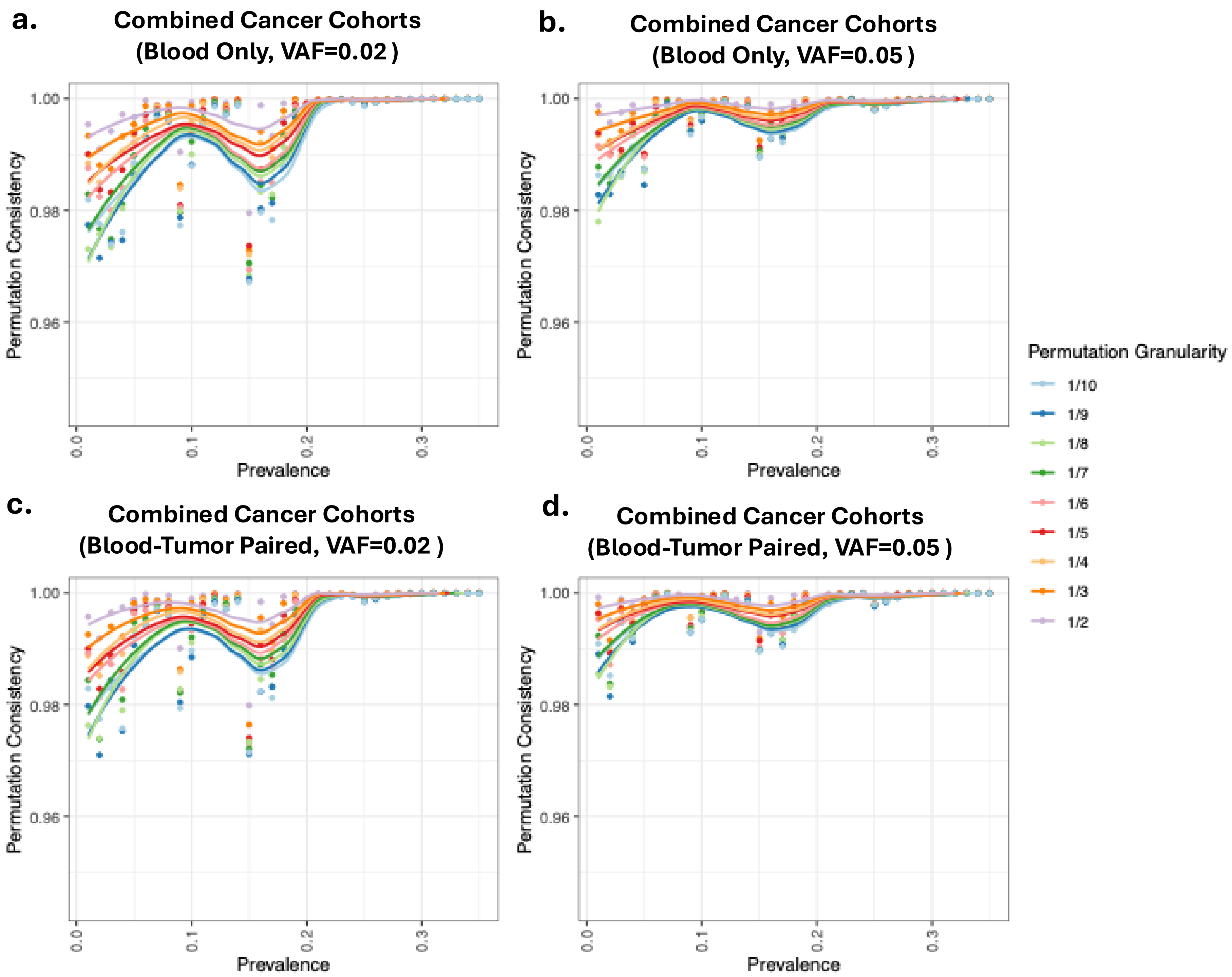

Supplementary Figure 11. Prevalence permutation analysis of combined ORIEN cancer cohorts (breast, colorectal, and lung). **a)**. Permutation consistency with blood only samples at VAF=0.02 and **b)**. VAF=0.05. **c)**. Permutation consistency with blood-tumor paired samples at VAF=0.02 and **d)**. VAF=0.05.

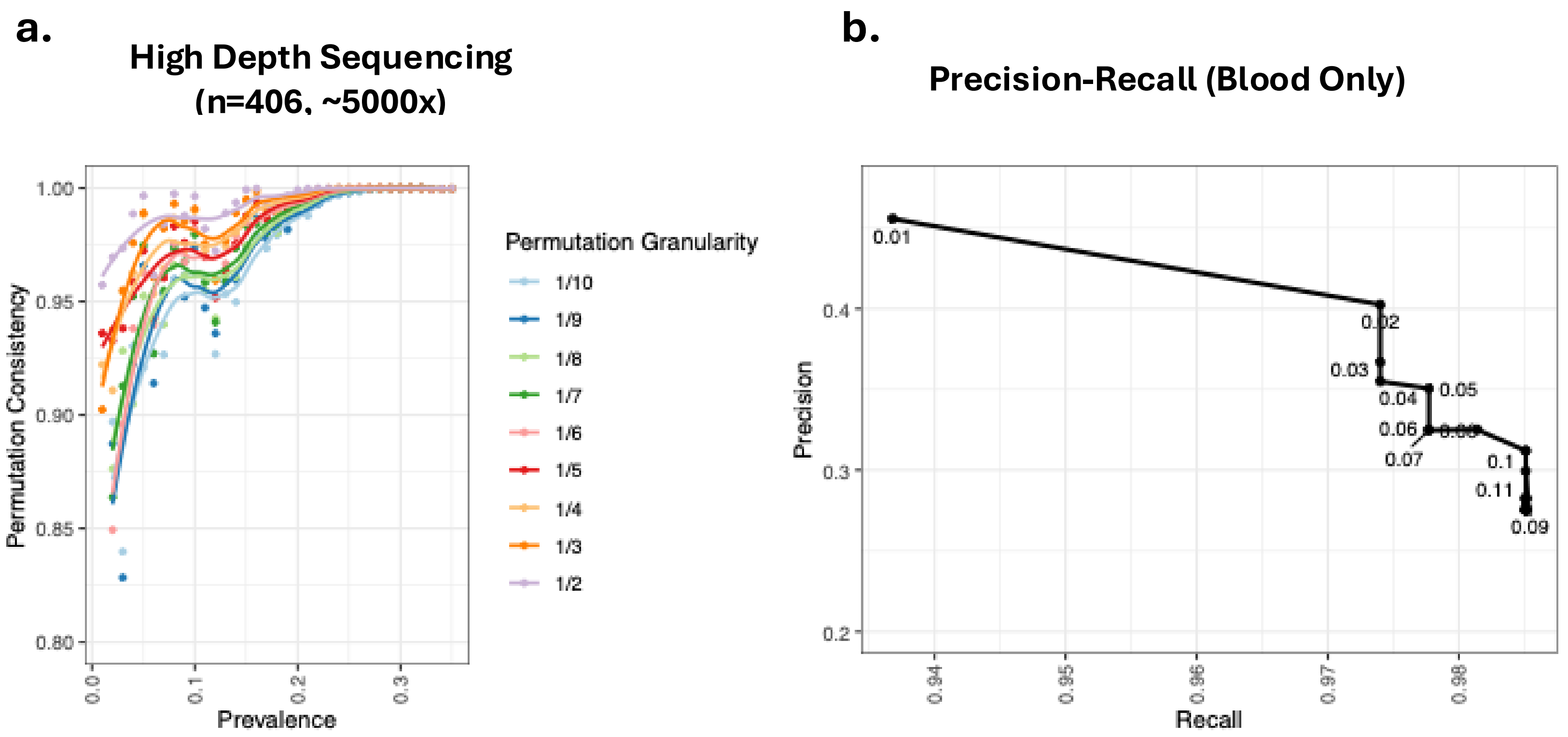

Supplementary Figure 12. Prevalence permutation analysis of blood samples from the ultra-high-depth sequencing breast cancer cohort. **a)**. Permutation consistency. **b)**. Precision and recall.

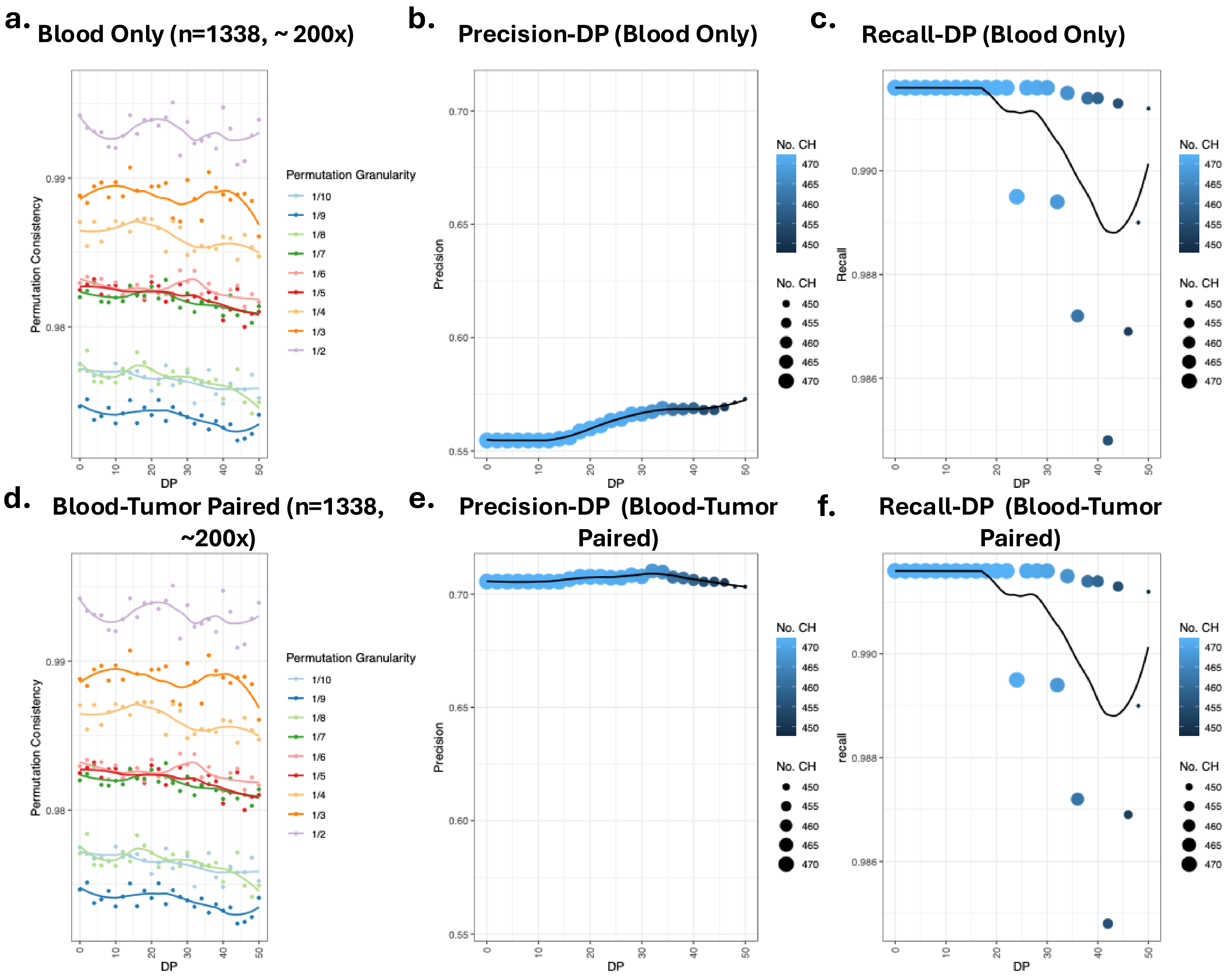

Supplementary Figure 13. DP permutation analysis of ORIEN breast cancer cohort. **a).** Permutation consistency, **b).** Precision, and **c).** Recall at different DP cutoffs using blood-only samples. **d).** Permutation consistency, **e).** Precision, and **f).** Recall at different DP cutoffs using blood-tumor paired samples.

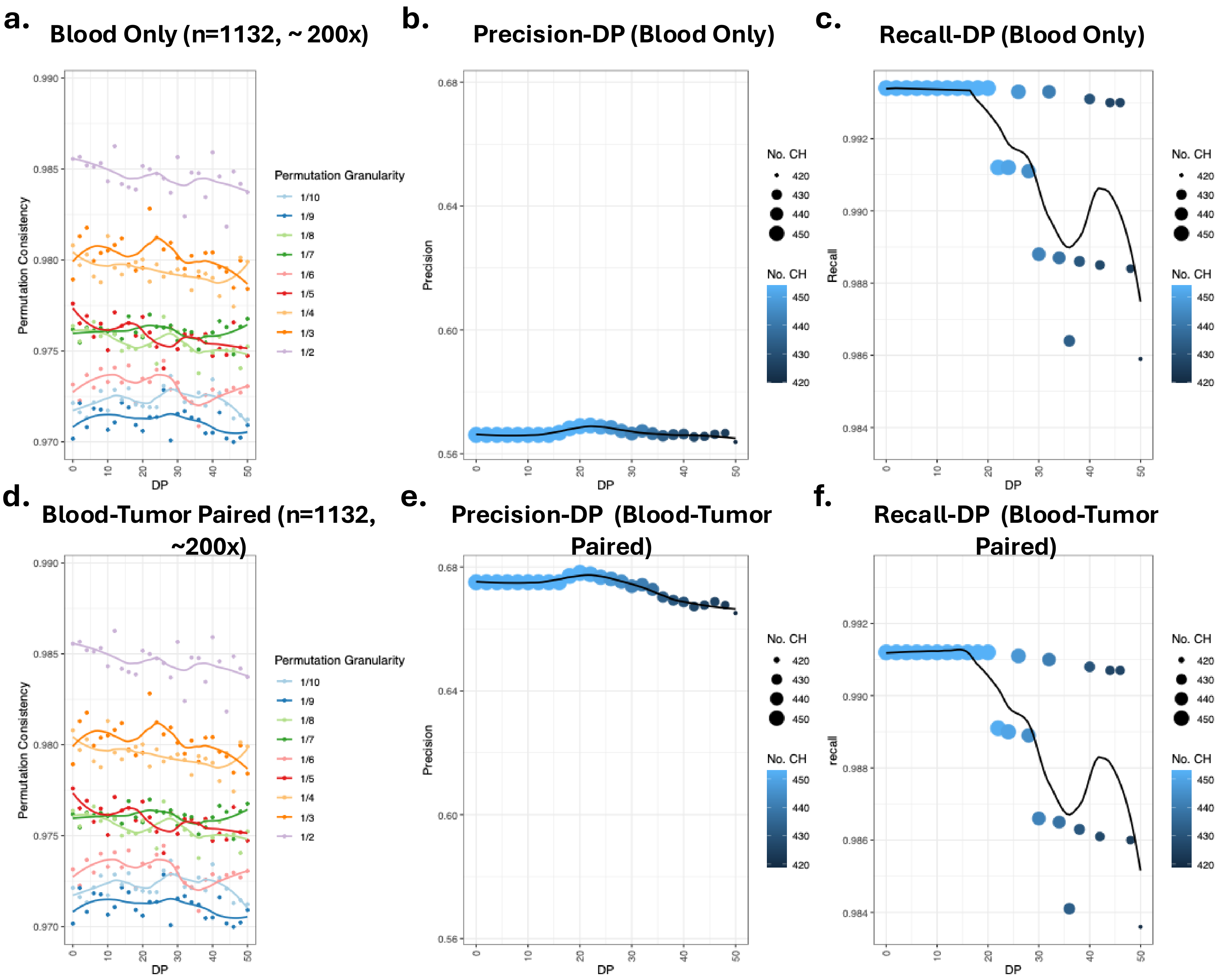

Supplementary Figure 14. DP permutation analysis of ORIEN colorectal cancer cohort. **a)**. Permutation consistency, **b)**. Precision, and **c)**. Recall at different DP cutoffs using blood-only samples. **d)**. Permutation consistency, **e)**. Precision, and **f)**. Recall at different DP cutoffs using blood-tumor paired samples.

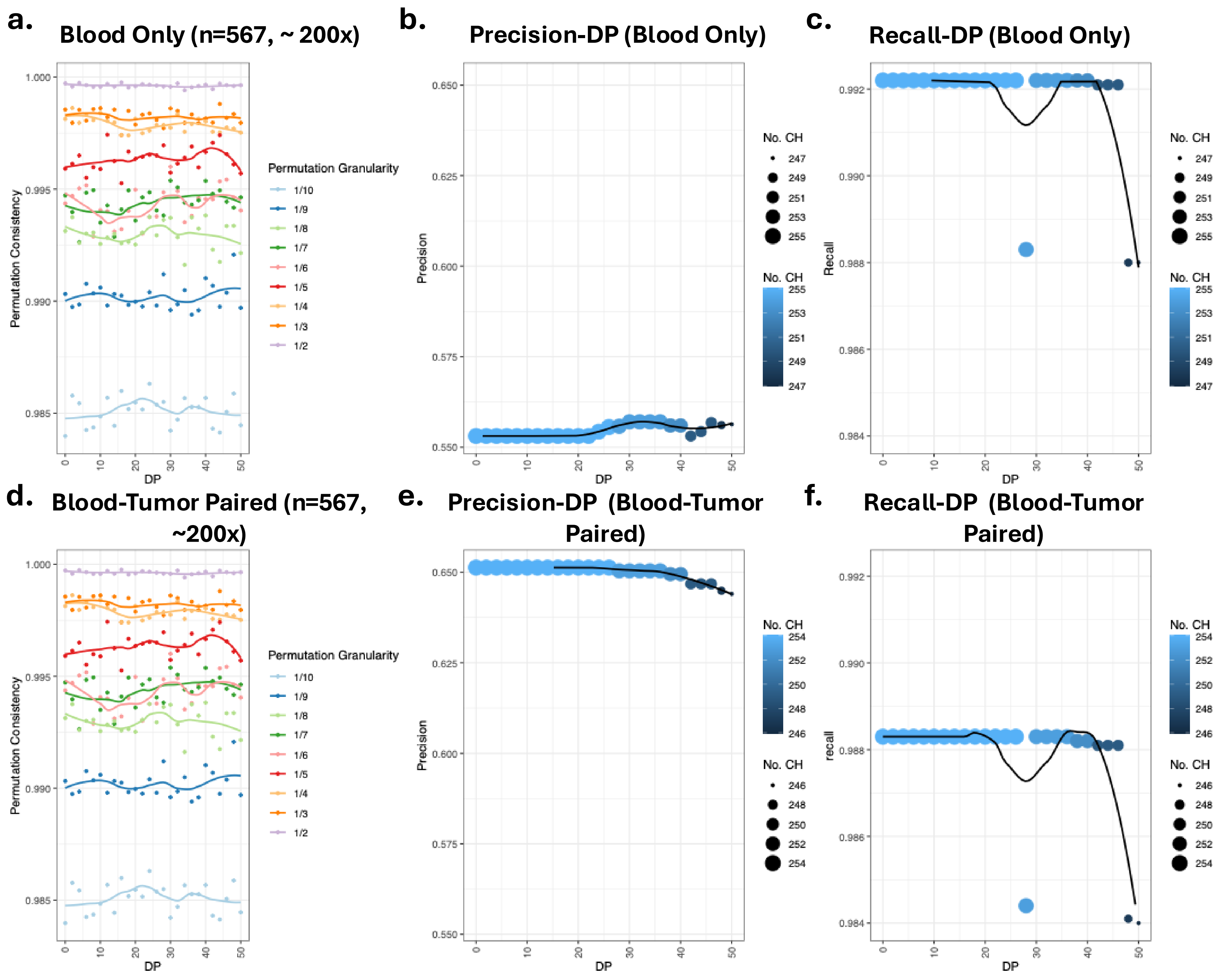

Supplementary Figure 15. DP permutation analysis of ORIEN lung cancer cohort. **a).** Permutation consistency, **b).** Precision, and **c).** Recall at different DP cutoffs using blood-only samples. **d).** Permutation consistency, **e).** Precision, and **f).** Recall at different DP cutoffs using blood-tumor paired samples.

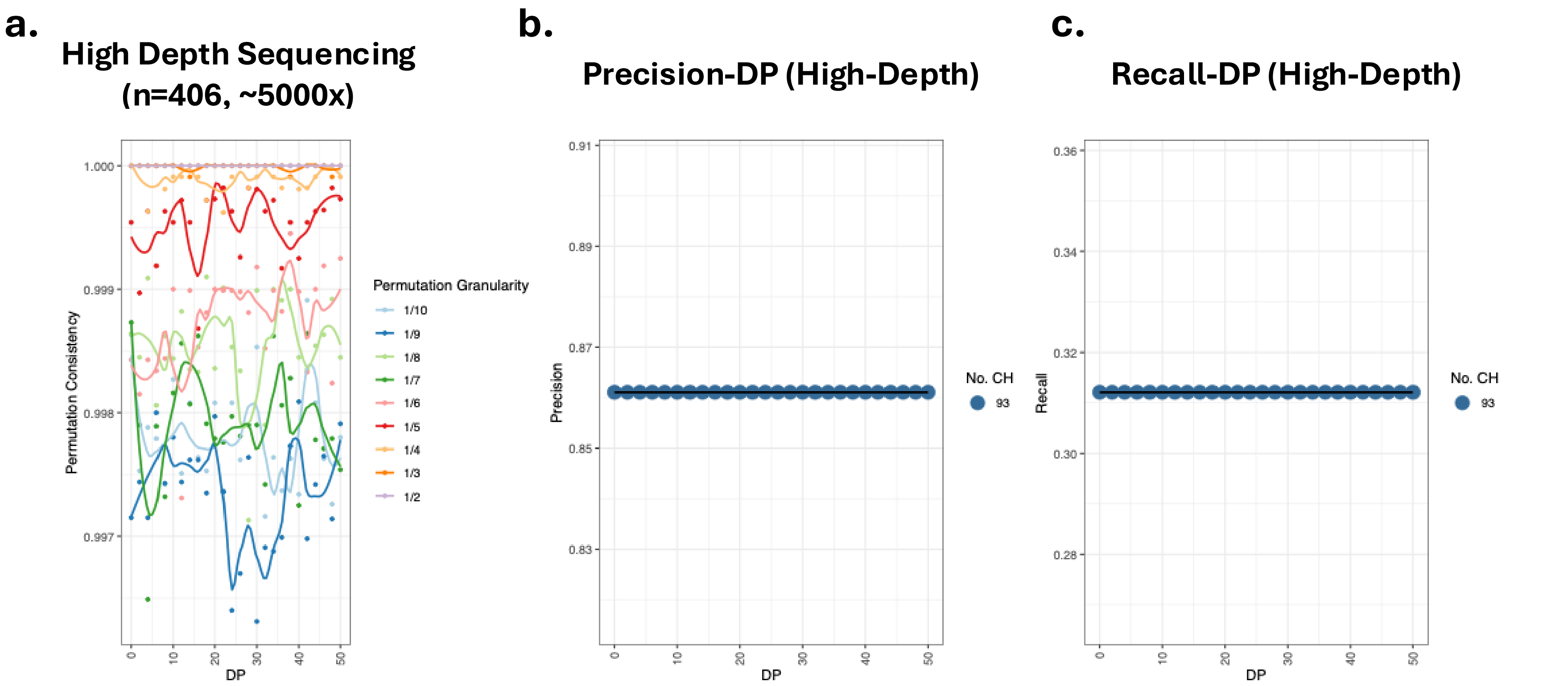

Supplementary Figure 16. DP permutation analysis of blood samples from the ultra-high-depth sequencing breast cancer cohort. **a)**. Permutation consistency. **b)**. Precision. **c)**. Recall.

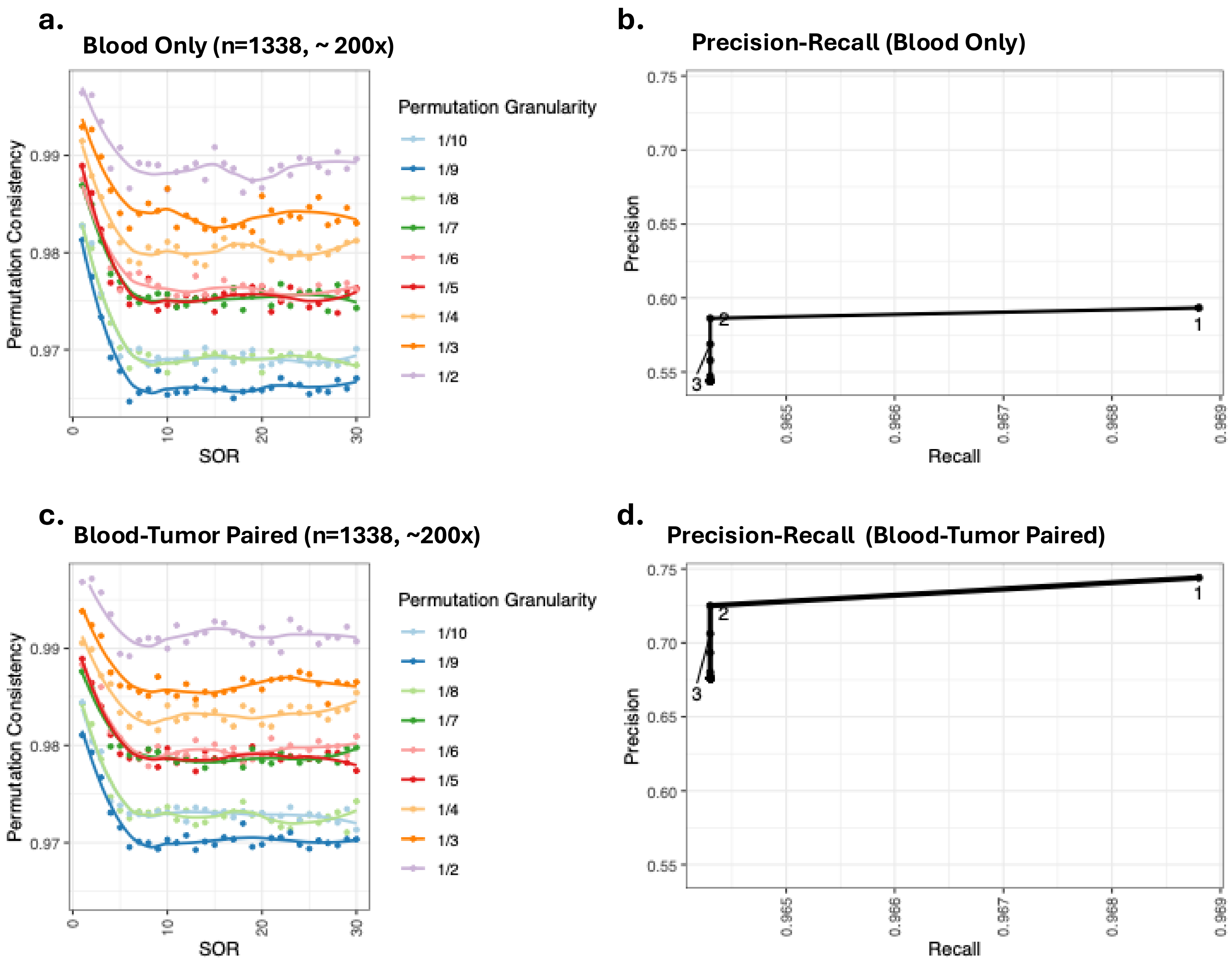

Supplementary Figure 17. SOR permutation analysis of ORIEN breast cancer cohort. **a)**. Permutation consistency and **b)**. Precision and recall at different SOR cutoffs using blood-only samples. **c).** Permutation consistency and **d).** Precision and recall at different SOR cutoffs using blood-tumor paired samples.

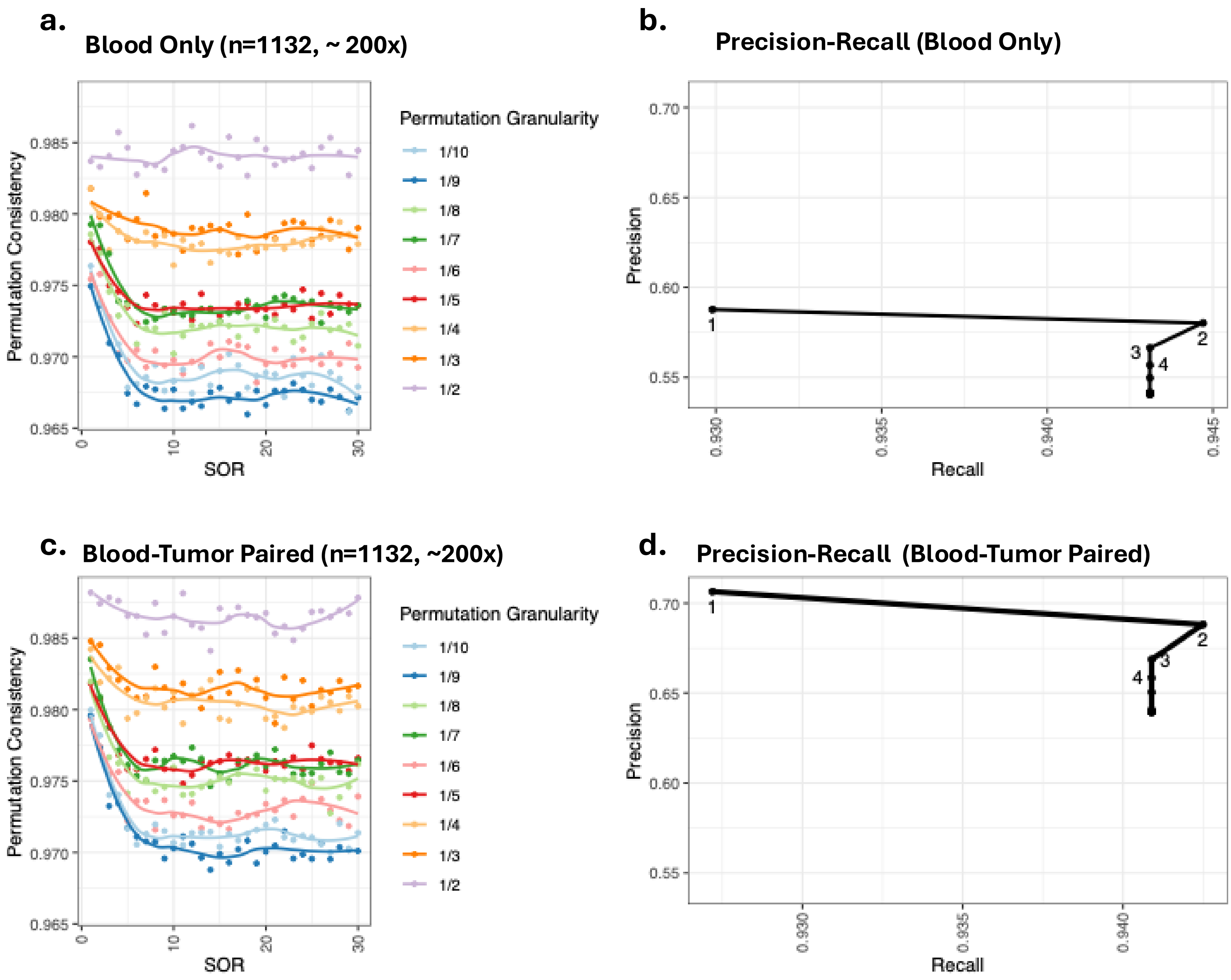

Supplementary Figure 18. SOR permutation analysis of ORIEN colorectal cancer cohort. **a)**. Permutation consistency and **b)**. Precision and recall at different SOR cutoffs using blood-only samples. **c).** Permutation consistency and **d).** Precision and recall at different SOR cutoffs using blood-tumor paired samples.

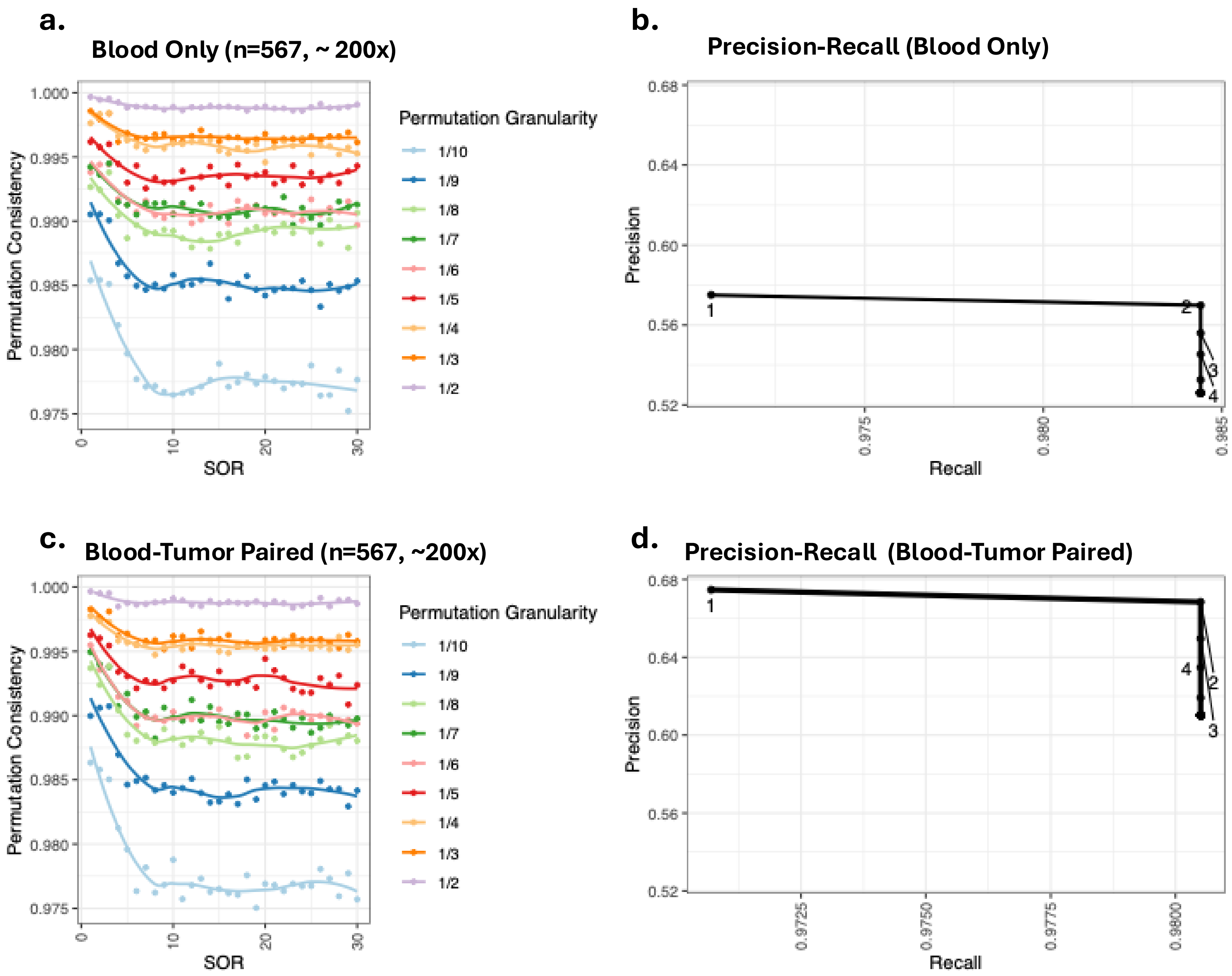

Supplementary Figure 19. SOR permutation analysis of ORIEN lung cancer cohort. **a)**. Permutation consistency and **b)**. Precision and recall at different SOR cutoffs using blood-only samples. **c).** Permutation consistency and **d).** Precision and recall at different SOR cutoffs using blood-tumor paired samples.

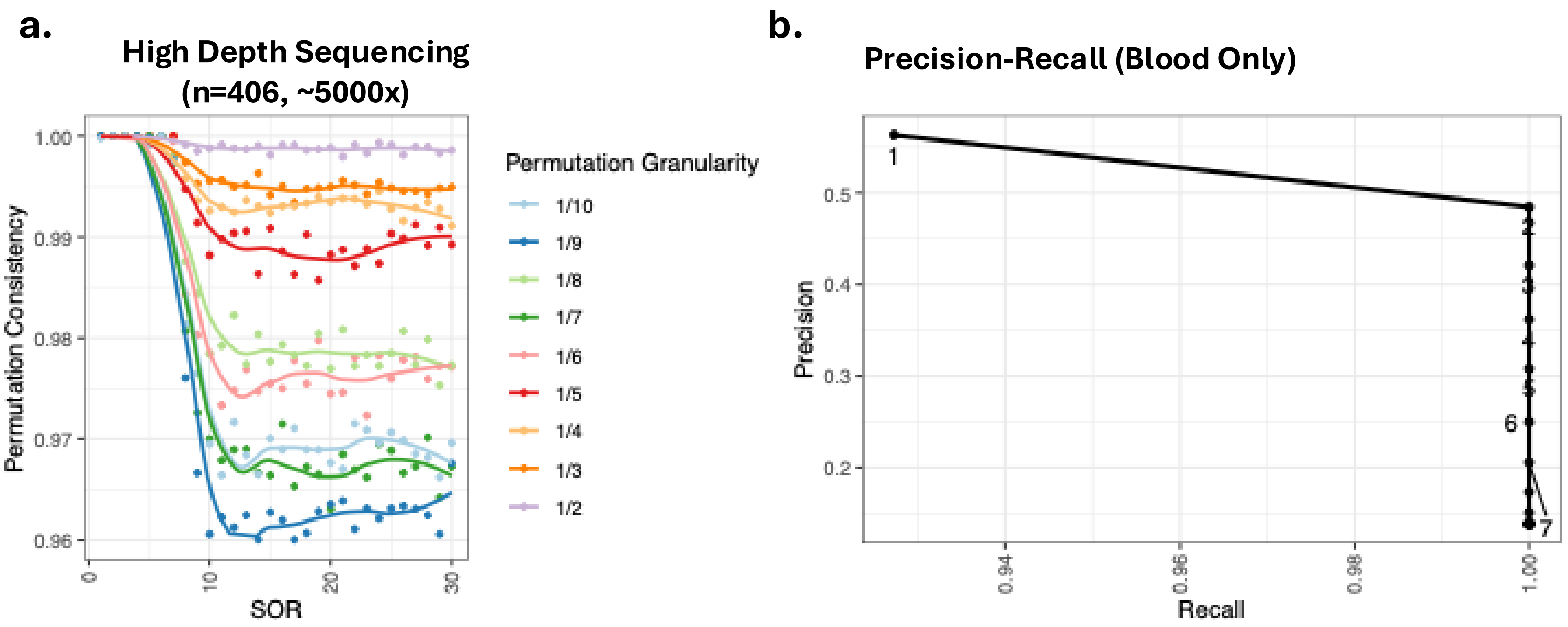

Supplementary Figure 20. SOR permutation analysis of blood samples from the ultra-high-depth sequencing breast cancer cohort. **a)**. Permutation consistency. **b)**. Precision and recall.

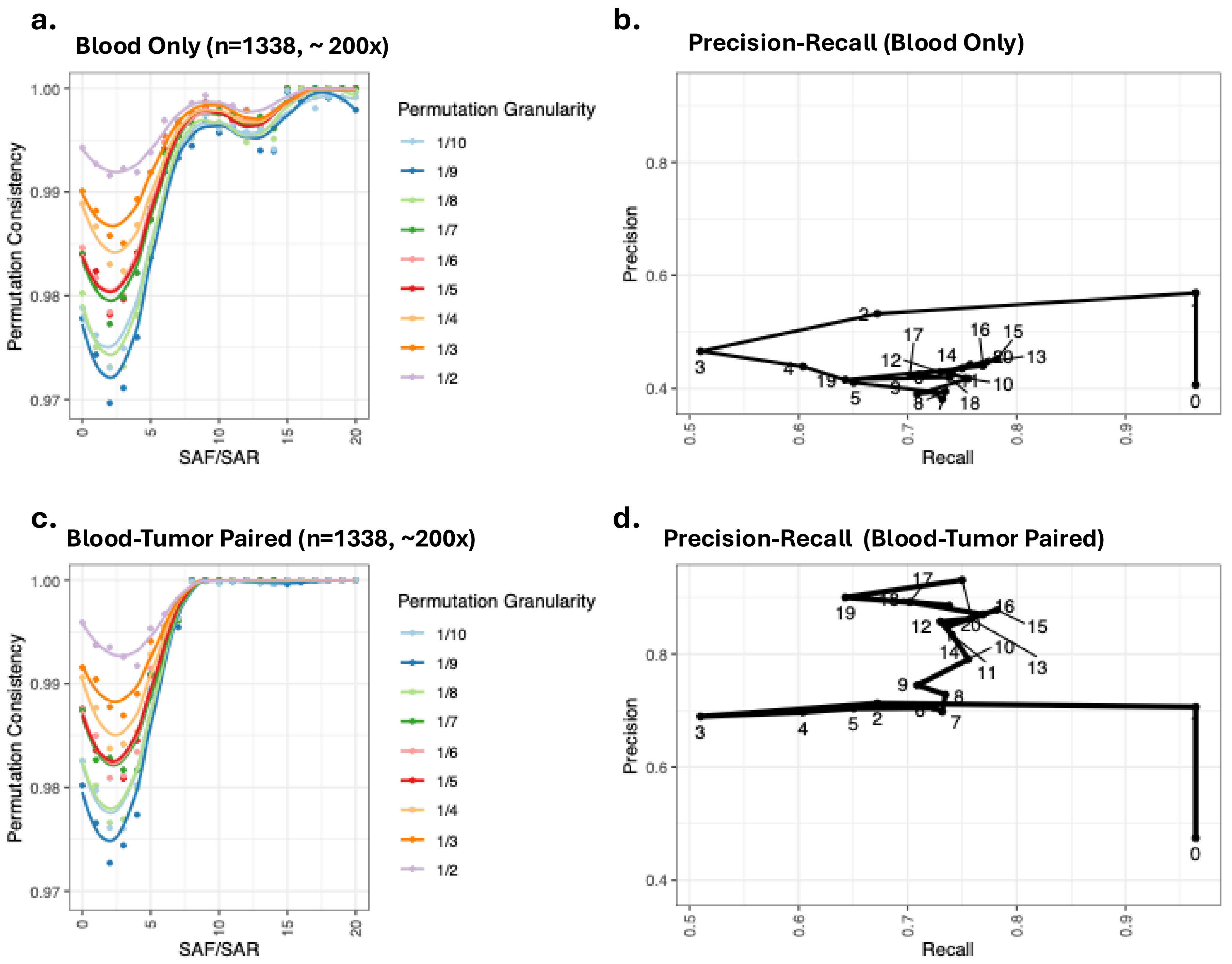

Supplementary Figure 21. SAF/SAR permutation analysis of ORIEN breast cancer cohort. **a)**. Permutation consistency and **b)**. Precision and recall at different SAF/SAR cutoffs using blood-only samples. **c).** Permutation consistency and **d).** Precision and recall at different SAF/SAR cutoffs using blood-tumor paired samples.

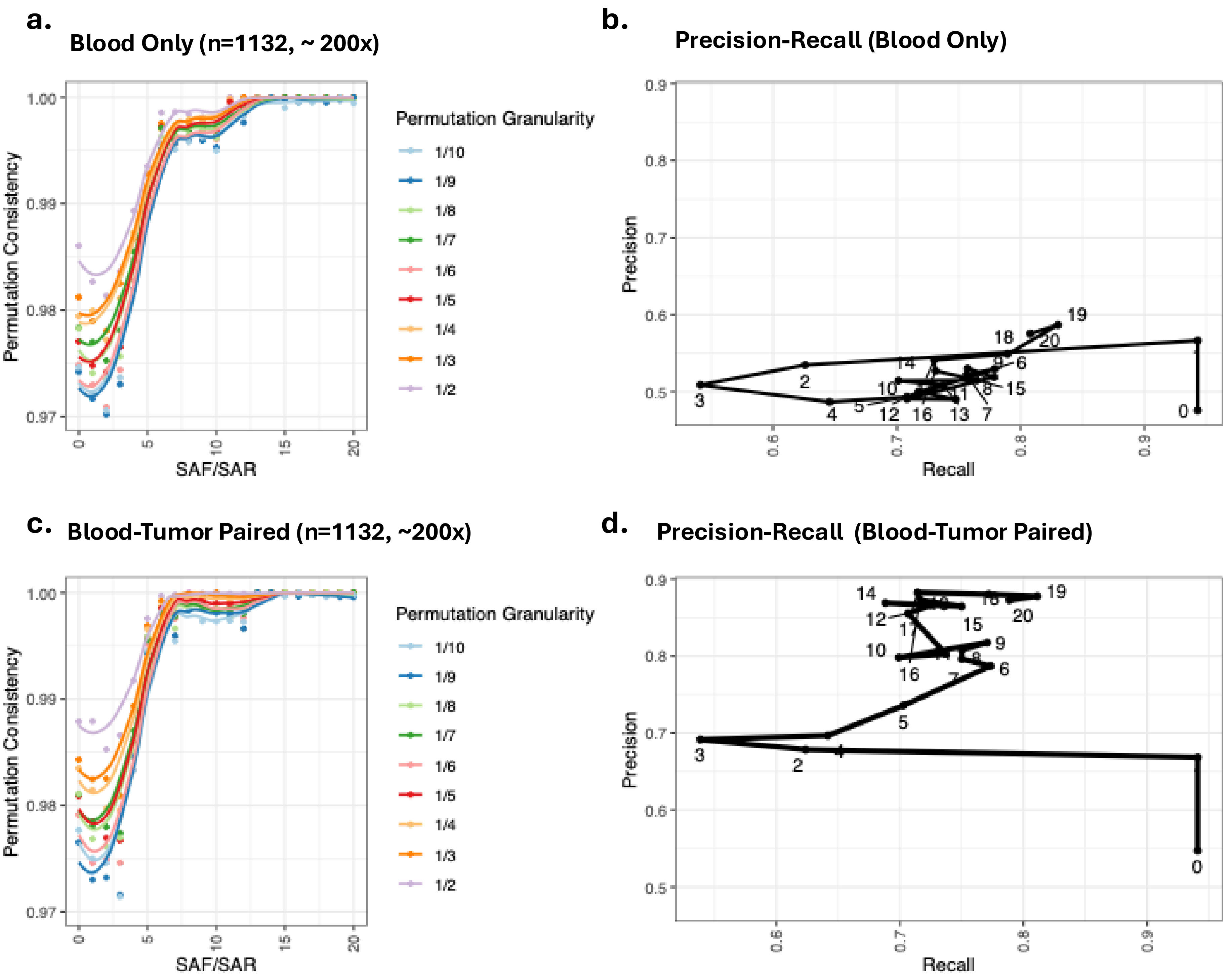

Supplementary Figure 22. SAF/SAR permutation analysis of ORIEN colorectal cancer cohort. **a)**. Permutation consistency and **b)**. Precision and recall at different SAF/SAR cutoffs using blood-only samples. **c).** Permutation consistency and **d).** Precision and recall at different SAF/SAR cutoffs using blood-tumor paired samples.

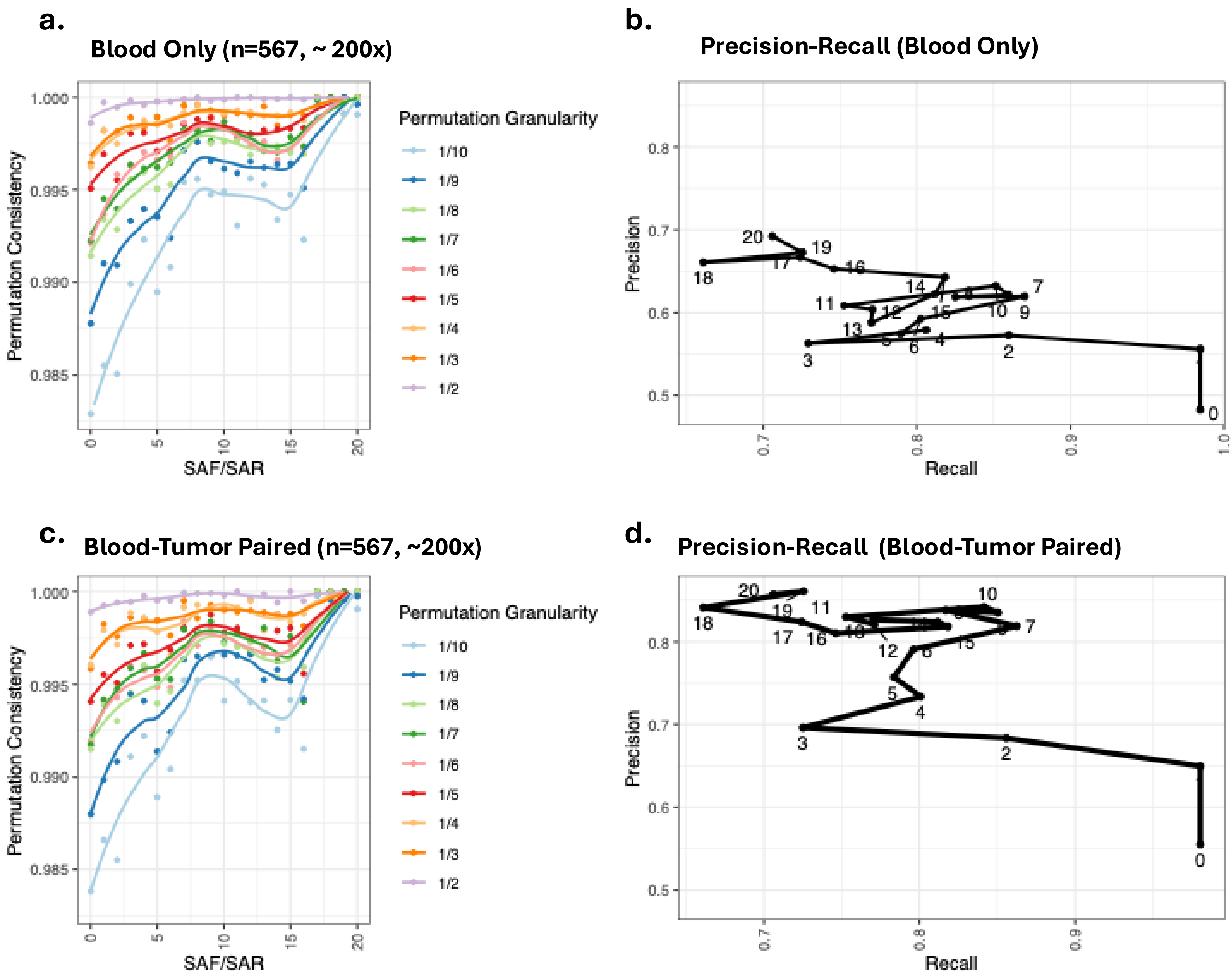

Supplementary Figure 23. SAF/SAR permutation analysis of ORIEN lung cancer cohort. **a)**. Permutation consistency and **b)**. Precision and recall at different SAF/SAR cutoffs using blood-only samples. **c)**. Permutation consistency and **d)**. Precision and recall at different SAF/SAR cutoffs using blood-tumor paired samples.

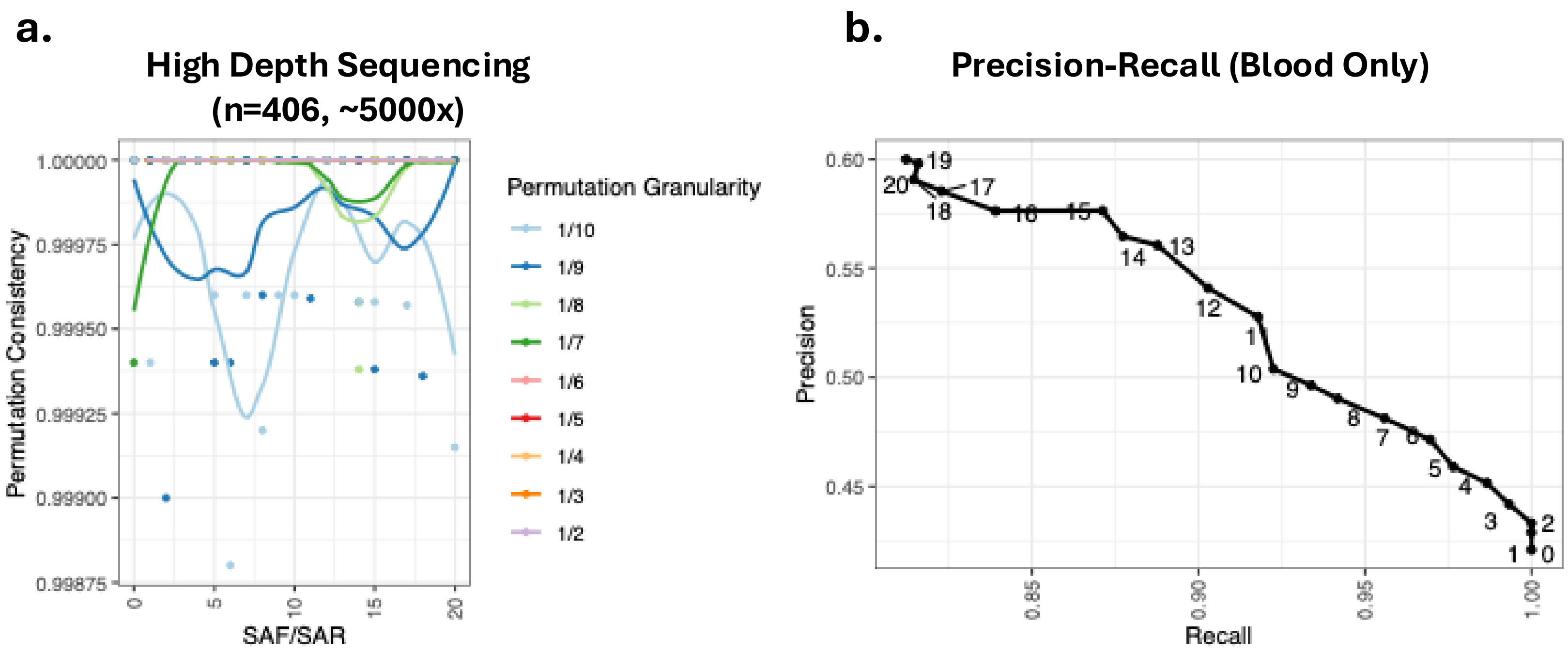

Supplementary Figure 24. SAF/SAR permutation analysis of blood samples from the ultra-high-depth sequencing breast cancer cohort. **a)**. Permutation consistency. **b)**. Precision and recall.

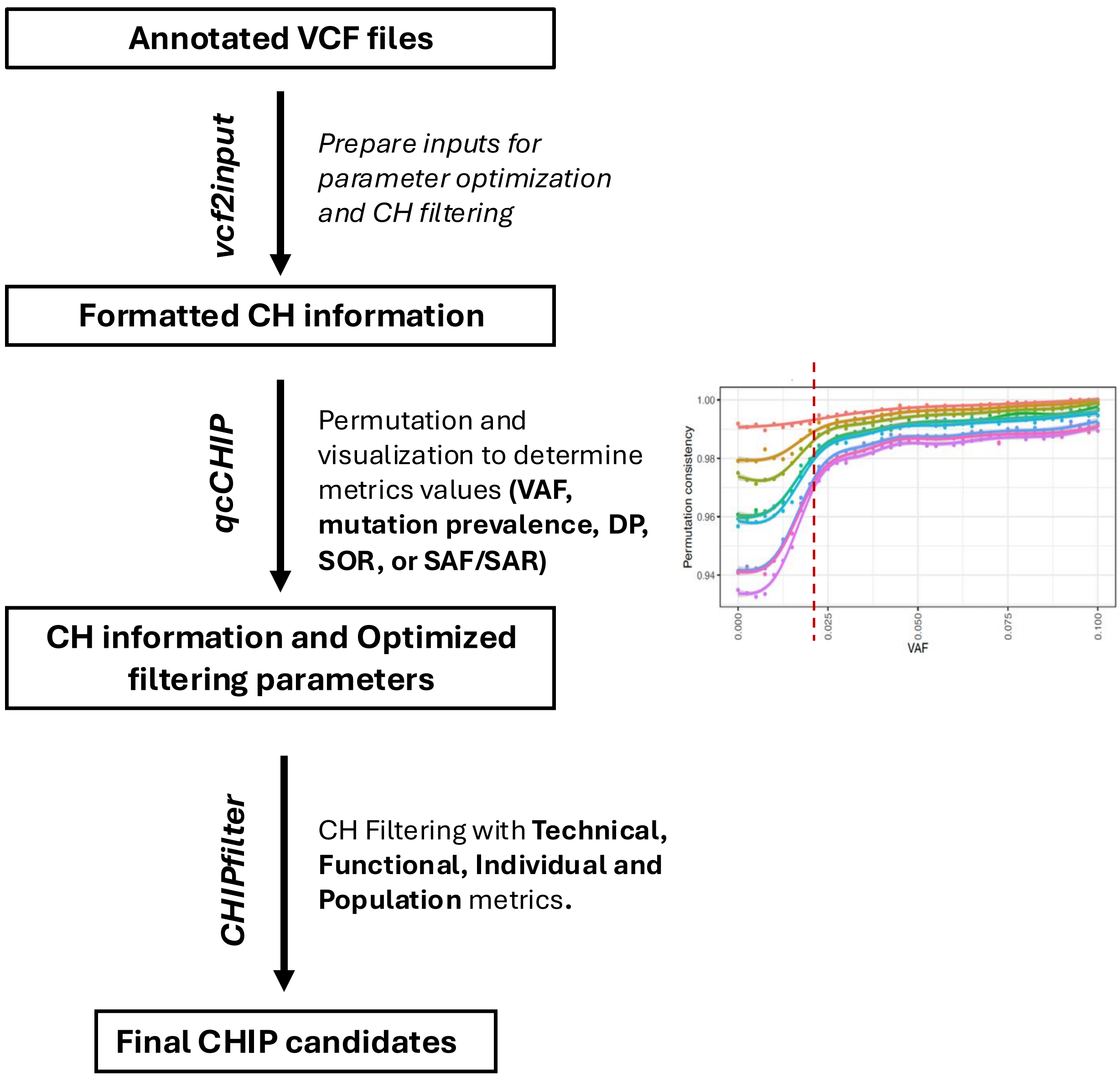

Supplementary Figure 25. Workflow of qcCHIP functions. First, *vcf2input* function uses annotated VCF files to generate the input file for downstream functions. Users can also directly provide a properly formatted table as shown in qcCHIP vignettes to skip this step. Then, *qcCHIP* function is used to determine values of five parameters (i.e. VAF, mutation prevalence, DP, SOR, or SAF/SAR) through premutation analysis of the studied cohort. Permutation consistency plots are generated at this step to guide the decision of optimal metric values. Users can also skip this step if they choose artificial metric values. Finally, *CHIPfilter* function is used to perform full quality filtering to identify CH mutations with chosen metric values.

Sample size	# of Subsets	Permuted Times	Time	Memory	# of CPUs	CPU Model
100	2	100	0:25:25	4.89Gb	12	AMD EPYC 75f3
100	5	100	0:31:36	9.98Gb	12	AMD EPYC 75f3
100	10	100	0:48:49	14.64Gb	12	AMD EPYC 75f3
500	2	100	0:31:27	3.74Gb	12	AMD EPYC 75f3
500	5	100	0:38:38	7.77Gb	12	AMD EPYC 75f3
500	10	100	0:47:16	11.92Gb	12	AMD EPYC 75f3
1000	2	100	0:39:22	3.87Gb	12	AMD EPYC 75f3
1000	5	100	0:39:31	8.08Gb	12	AMD EPYC 75f3
1000	10	100	0:55:15	12.03Gb	12	AMD EPYC 75f3

Supplementary Figure 26. Time and memory costs of permutations by qcCHIP.
